# Purely Spin‐Vibronic Coupling Assisted Triplet to Singlet Up‐Conversion for Real Deep Blue Organic Light‐Emitting Diodes with Over 20% Efficiency and y Color Coordinate of 0.05

**DOI:** 10.1002/advs.202101137

**Published:** 2021-08-13

**Authors:** Vilas Venunath Patil, Ha Lim Lee, Inkoo Kim, Kyung Hyung Lee, Won Jae Chung, Joonghyuk Kim, Sangho Park, Hyeonho Choi, Won‐Joon Son, Soon Ok Jeon, Jun Yeob Lee

**Affiliations:** ^1^ School of Chemical Engineering Sungkyunkwan University Suwon 16419 Korea; ^2^ Data and Information Technology Center Samsung Electronics Hwaseong 18448 Korea; ^3^ Samsung Advanced Institute of Technology Samsung Electronics Suwon 16678 Korea

**Keywords:** blue organic light‐emitting diodes, efficiency, narrow full‐width‐at‐half‐maximum, spin–vibronic coupling, Stokes shift

## Abstract

Finding narrow‐band, ultrapure blue thermally activated delayed fluorescence (TADF) materials is extremely important for developing highly efficient organic light‐emitting diodes (OLEDs). Here, spin–vibronic coupling (SVC)‐assisted ultrapure blue emitters obtained by joining two carbazole‐derived moieties at a *para* position of a phenyl unit and performing substitutions using several blocking groups are presented. Despite a relatively large singlet–triplet gap (∆*E*_ST_) of >0.2 eV, efficient triplet‐to‐singlet crossover can be realized, with assistance from resonant SVC. To enhance the spin crossover, electronic energy levels are fine‐tuned, thereby causing ∆*E*_ST_ to be in resonance with a triplet–triplet gap (∆*E*_TT_). A sizable population transfer between spin multiplicities (>10^3^ s^−1^) is achieved, and this result agrees well with theoretical predictions. An OLED fabricated using a multiple‐resonance‐type SVC‐TADF emitter with CIE color coordinates of (0.15, 0.05) exhibits ultrapure blue emissions, with a narrow full‐width‐at‐half‐maximum of 21 nm and a high external quantum efficiency of 23.1%.

## Graphical abstract

Spin‐vibronic coupling assisted ultrapure blue emitters demonstrate high external quantum efficiency of 23.1%, color coordinate of (0.15,0.05) and small full width at half maximum of 21 nm.

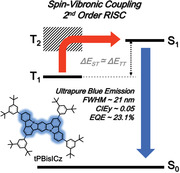

## 1 Introduction

According to spin statistics obtained under electrical excitation,^[^ ^1^ ^]^ the generation of non‐radiative triplet excitons is thrice that of radiative singlet excitons. Therefore, a major challenge in designing organic light‐emitting diode (OLED) emitters that can achieve 100% internal quantum efficiency (IQE) has been the conversion of both the types of excitons to radiative photons.^[^ ^2^ ^]^ Facile intra‐molecular lowest‐energy triplet–singlet (T_1_–S_1_) inter‐conversion is one potential solution to this problem. Thermally activated delayed fluorescence (TADF), which permits 100% IQE of singlet emission through triplet‐to‐singlet reverse inter‐system crossing (RISC), has emerged as a promising alternative to the more traditional phosphorescence, which is mediated by the large spin–orbit coupling (SOC) of precious metals.^[^ ^3^ ^]^ Naturally, a decisive parameter governing TADF performance is the S_1_–T_1_ energy gap (∆*E*_ST_), which, according to a simple two‐state model,^[^ ^4^ ^]^ is related to the exchange interaction *K* between electrons in the highest‐occupied molecular orbital (HOMO) and the lowest‐unoccupied molecular orbital (LUMO) according to the relationship ∆*E*_ST_ ≃ 2*K*.

Nascent TADF emitters adopted a donor–acceptor (DA)‐type molecular design aimed at spatially separating the frontier orbitals because in such molecules, the HOMO and LUMO reside on the donor and acceptor moieties, respectively, thereby reducing *K* and ∆*E*_ST_.^[^ ^5^ ^]^ Although a small ∆*E*_ST_ of <0.1 eV and a high RISC rate (*k*_RISC_) of the order of 10^6^ s^−1^ can be realized, the unavoidable charge‐transfer character of the emissive S_1_ state generates a broad‐band emission feature, resulting in low color purity, typically with a full‐width‐at‐half‐maximum (FWHM) of >50 nm.^[^ ^6^ ^]^ Thus, for display applications, a narrow‐band color filter needs to be coupled to selectively transmit a primary color, thus impairing the light extraction efficiency. A recently developed molecular design concept based on the multiple‐resonance (MR) effect has shown great potential for the remediation of such a broad emission band by judiciously placing electron‐donating atoms and electron‐accepting atoms in such a manner that the distributions of the frontier orbitals alternate within a rigid *π*‐conjugation scaffold to realize a short‐range charge transfer (CT). Consequently, in MR‐TADF emitters, a narrow‐banded spectrum with FWHM <30 nm can be simultaneously achieved along with reduced ∆*E*_ST_.

The design concepts of conventional MR‐TADF emitters proposed so far require a combination of electron‐deficient and electron‐rich atoms such as boron–nitrogen (B/N),^[^ ^7^, ^8^, ^9^, ^10^, ^11^ ^]^ boron–oxygen,^[^ ^12^, ^13^ ^]^ or carbonyl–nitrogen^[^ ^14^, ^15^ ^]^ within a polycyclic aromatic hydrocarbon (PAH) framework.^[^ ^16^ ^]^ However, none of these PAH‐type fluorescent emitters has achieved ultrapure blue color with a *y*‐color coordinate of 0.05 corresponding to the BT.2020 color standard established by the International Telecommunication Union.^[^ ^17^ ^]^ 5,9‐Diphenyl‐5,9‐diaza‐13b‐boranaphtho[3,2,1‐de]anthracene (DABNA‐1), which is a representative PAH material, offers an external quantum efficiency (EQE) of 13.5% with CIE color coordinates of (0.13, 0.09).^[^ ^7^ ^]^ The linear extension of *π*‐conjugation aids in improving efficiencies, but color purity is often compromised by red‐shifted emission wavelengths.^[^ ^9^ ^]^ *N*,*N*‐dimesityl‐9,9‐dimethyl‐5,5‐diphenyl‐5,9‐dihydroquinolino[3,2,1‐de]acridin‐3‐amine (DMACN‐B) showing maximum EQE of 10% with CIE color coordinates of (0.151, 0.045) was also reported but still suffered from low EQE.^[^ ^18^ ^]^ Therefore, the discovery of ultrapure blue emitters that have a novel core structure and can offer CIE*_y_* ≈ 0.05 and a theoretical maximum EQE of 20% (assuming an out‐coupling efficiency of 20%) is a major challenge in the development of high‐efficiency deep‐blue OLEDs. Recently, we developed a narrow‐band violet fluorescent emitter with CIE*_y_* = 0.018 and FWHM = 14 nm by fusing indolo[3,2,1‐jk]carbazole (ICz) with a carbazole unit and orienting the nitrogen atoms *meta* to the anchoring phenyl ring.^[^ ^19^ ^]^ The emissive singlet exhibited the characteristics of an MR structure; however, the huge ∆*E*_ST_ (as large as 0.44 eV) prevented the occurrence of TADF entirely, resulting in poor efficiency.

Here, we present spin–vibronic coupling (SVC)‐assisted ultrapure blue TADF emitters based on indolo[3,2,1‐jk]indolo[1′,2′,3′:1,7]indolo[3,2‐b]carbazole (BisICz), which was constructed by fusing an ICz unit with a carbazole unit through *para*‐oriented nitrogen atoms. Both the S_1_ and T_1_ states of bare BisICz showed significant MR characteristics, and a reduced ∆*E*_ST_ of approximately 0.3 eV was obtained from higher‐level quantum chemical calculations and photophysical characterizations. Because of the non‐negligibly large ∆*E*_ST_ and insignificant S_1_–T_1_ SOC, the direct T_1_→S_1_ population transfer was intrinsically hindered, and the second‐order SVC played a crucial role in the spin crossover and even provided unique resonance conditions that enhanced RISC dynamics.^[^ ^20^, ^21^ ^]^ We demonstrated that the electronic energy levels could be fine‐tuned by incorporating electron‐donating groups into appropriate substitution sites on BisICz to induce resonant SVC and thereby achieve efficient RISC. Ultimately, we obtained new design principles for a high‐efficiency deep‐blue TADF emitter, which had an unprecedented CIE of (0.15, 0.05), a high EQE of 23.1%, and a small FWHM of 21 nm.

## 2 Results and Discussion

### 2.1 Molecular Design

**Figure 1:**
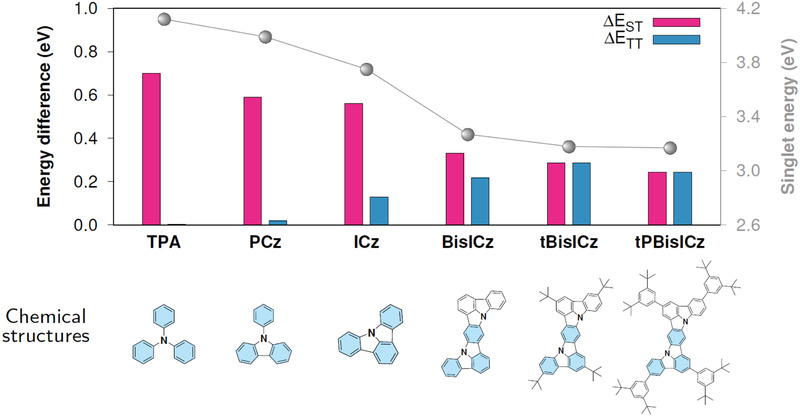
Molecular design. Chemical structures, energy differences, ∆*E*_ST_ (adiabatic), ∆*E*_TT_ (vertical), and singlet energy (vertical) of each molecule. The TPA cores are shaded in skyblue. ∆*E*_ST_ and the singlet energy were calculated at the SCS‐ADC(2) level, whereas ∆*E*_TT_ was calculated at the *ω* ^∗^B97M‐V level. The vertical energies were evaluated for the S_1_ geometry.

We started with tri‐phenyl amine (TPA) and constructed phenyl carbazole (PCz) and ICz by successively binding the adjacent phenyl rings into a planar structure (**Figure** **1**). We employed a suitably high level of theory, SCS‐ADC(2)/def2‐SVP, which includes electron correlations in the form of double excitations to correctly predict energies and electron densities of excited states.^[^ ^22^, ^23^ ^]^ In this planarization of TPA, a decrease in ∆*E*_ST_ corroborates well with an increase in the MR effect in the S_1_ state upon S_0_–S_1_ excitation by the alternating distributions of hole and particle densities, as shown in the difference density plot in Figure S1, Supporting Information. However, owing to the predominant local excitation of the *π*–*π* ^∗^ character of the T_1_ state, whose level is stabilized by a large *K*, ∆*E*_ST_ remains significantly high (>0.5 eV), which prevents the realization of any meaningful RISC process. Moreover, even for ICz, the predicted wavelengths of singlet emission appear to be too short for it to be used as a blue emitter.

For triplet harvesting via RISC, not only should the S_1_ energy for ultrapure blue emission be reduced without disrupting the MR effect but the MR effect should also be sustained in the T_1_ state for a small ∆*E*_ST_. Therefore, we devised a new core named BisICz by fusing an ICz unit with a carbazole unit via the orientation of the nitrogen atoms *para* to the anchoring phenyl ring. In the case of the structural isomer of BisICz, wherein the nitrogen atoms were oriented to *meta* positions, a large ∆*E*_ST_ of 0.44 eV was predicted because of the persistent *π*–*π* ^∗^ character in the T_1_ state (Figure S2, Supporting Information). This result is in agreement with that of our previous experiment where no TADF activity was observed for 2,5,13,16‐tetra‐*tert*‐butylindolo[3,2,1‐jk]indolo[1′,2′,3′:1,7]indolo[2,3‐b]carbazole (tDIDCz).^[^ ^19^ ^]^ In BisICz, although ∆*E*_ST_ decreases to 0.33 eV compared to 0.56 eV for ICz, efficient spin crossover still remains a difficult challenge because the vibrational resonance mechanism of MR‐TADF emitters operates at ∆*E*_ST_ <0.2 eV, and hence, newer molecular design approaches are clearly needed. The existence of the close‐lying T_2_ state with the T_1_–T_2_ energy gap (∆*E*_TT_) of 0.22 eV opens the possibility of engineering the energy levels required to optimize the resonance condition necessary for SVC‐TADF emitters. Specifically, ∆*E*_ST_ of BisICz should be reduced further by ≈0.1 eV.

Using quantum chemical methods, we performed a cursory survey on the substituent effect at the 3,6‐positions of an isolated carbazole, which are well‐known sites that affect the HOMO level and increase electrochemical stabilities.^[^ ^24^ ^]^ Accordingly, two substituents were identified to have the ability to reduce ∆*E*_ST_. The addition of an electron‐donating *tert*‐butyl group to these sites destabilized the HOMO, whereas the addition of the phenyl group of a similar electron donor afforded the stabilization of the LUMO through the delocalization of a nitrogen lone pair (Table S1, Supporting Information).

Thus, we designed and synthesized three ultrapure blue emitters: BisICz, 2,5,11,14‐tetrakis(1,1‐dimethylethyl)indolo[3,2,1‐jk]indolo[1′,2′,3′:1,7]indolo[3,2‐b]carbazole (tBisICz), and 2,5,11,14‐tetrakis(3,5‐di‐tert‐butylphenyl)‐indolo[3,2,1‐jk]indolo[1′,2′,3′:1,7]indolo[3,2‐b]carbazole (tPBisICz) (Scheme S1, Supporting Information). Instead of adopting bare phenyl groups, we used 3,5‐di‐*tert*‐butylphenyl groups to minimize undesirable molecular packing in the amorphous solid state encountered in the emissive layer of OLED. For a rigid narrow‐band MR emitter with a large ∆*E*_ST_, achieving a small energy denominator associated with an on‐resonance spin–vibronic (SV) model (Equation (2)) is essential for opening SVC‐TADF materials for triplet harvesting. The energy denominator ∆*E*_TT_ − ∆*E*_ST_ for our emitters followed the order of BisICz (113 meV) > tBisICz (26 meV) > tPBisICz (6 meV). Based on these values, the latter two were considered to be suitable candidates for SVC‐TADF emitters (Table S2, Supporting Information).

### 2.2 Resonant SVC

From a more fundamental perspective, the SV enhancement in the T_1_–S_1_ crossover is a second‐order effect of an interaction Hamiltonian, which simultaneously considers the spin–orbit operator *H*ˆSO and non‐Born–Oppenheimer operator *H*ˆnBO, thereby introducing the possibility of a strong inter‐state vibronic coupling. The coupling matrix element up to the second order can be expressed as^[^ ^25^, ^26^ ^]^

(1) $$H_{\mathrm{ST}}=\underset{\mathrm{DSO}}{\underset{︸}{H_{\mathrm{ST}}^{\mathrm{SO}}}}+\frac{1}{2}\sum_{T^{\prime }}H_{{\mathrm{ST}}^{\prime }}^{\mathrm{SO}}H_{T^{\prime }T}^{\mathrm{nBO}}\left(\frac{1}{E_{T^{\prime }}-E_{T}}+\frac{1}{E_{T^{\prime }}-E_{S}}\right)$$

We immediately recognized two types of spin crossover interactions: the direct spin–orbit (DSO) coupling, which is governed by the electronic characters of the involved states, and the SVC, which interacts with the SOC between the singlet and triplet states within the triplet manifold. To ensure pragmatic clarity in the following discussion on particular resonance conditions, we have deliberately excluded an additional SV term with mediating states in the singlet manifold. However, this term is considered in our quantitative analysis on RISC dynamics. We have also omitted index 1 of the S_1_ and T_1_ states for simplicity.

**Figure 2:**
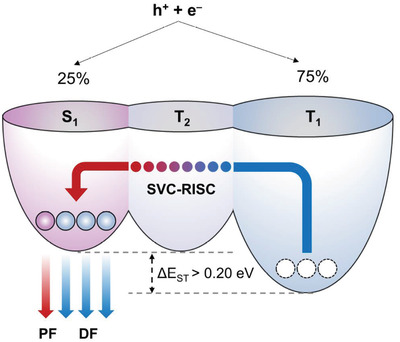
SVC‐TADF mechanism. Schematic diagram of triplet harvesting of electro‐generated excitons by TADF via on‐resonance SVC. h^+^, hole; e^−^, electron; PF, prompt fluorescence; DF, delayed fluorescence; RISC, reverse inter‐system crossing.

**Figure** **2** illustrates a viable SV RISC pathway enhanced by resonance conditions for SVC‐TADF emitters. In MR‐TADF emitters, the S_1_ and T_1_ states have an identical electronic nature, which can be described as a short‐range CT, and according to the El‐Sayed rule,^[^ ^27^ ^]^ the SOC between these states is insignificant. A recent work elucidated the RISC mechanism behind the excellent B/N‐configuration emitters^[^ ^28^ ^]^: when the frequency of a vibration‐inducing non‐adiabatic coupling (NAC) between T_1_ and higher‐lying T_n_ states matches ∆*E*_ST_, an SVC can accelerate the RISC process dramatically. The successive and continuous occurrence of such vibrational modes necessitates a resonantly enhanced RISC over a wide range of ∆*E*_ST_. Nevertheless, such resonance enhancement is inevitably terminated by the absence of vibrational modes >0.2 eV, except in the case of hydrogen stretching modes, which exhibit negligible NAC. Although emitters with large gaps (>0.2 eV) are not very promising, they can, in principle, be brought under resonance for second‐order RISC by utilizing the energetic resonance between the T_2_ and S_1_ states based on the SVC expressed in Equation (1). The T_2_ state then provides the largest contribution to the sum‐over‐states expansion, and Equation (1) can be largely approximated as

(2) $$H_{S_{1}T_{1}}≃\frac{1}{2}\frac{H_{S_{1}T_{2}}^{\mathrm{SO}}H_{T_{2}T_{1}}^{\mathrm{nBO}}}{\Delta E_{\mathrm{TT}}-\Delta E_{\mathrm{ST}}}$$

where ∆*E*_TT_ = *E*_T2_ − *E*_T1_. The above resonance condition requires a novel molecular core that is different from that of conventional B/N analogues because the succeeding T_2_ state is often found to be significantly higher in energy than the S_1_ state; for instance, ∆*E*_TT_ = 0.59 eV for DABNA‐1.^[^ ^24^ ^]^ Therefore, the resonance condition in Equation (2) cannot be employed. This resonance condition is also impractical for DA‐type molecules. For instance, when the nearly degenerate CT states are higher in energy than the^3^LE state, the above resonance condition may nearly be satisfied. However, the resonance enhancement vanishes simply because of the small SOC between the CT states, and instead, RISC occurs through the El‐Sayed‐allowed direct SOC between the ^1^CT and ^3^LE states if ∆*E*_ST_ is sufficiently small.

### 2.3 Photophysical Properties

Table S3, Supporting Information, summarizes the measured and calculated photophysical properties of BisICz‐based compounds. Photophysical measurements were performed in diluted dichloromethane and in a thin film state using a high‐triplet‐energy mixed host, that is, 1,3‐di(9H‐carbazol‐9‐yl)benzene (mCP):diphenyl(4‐(triphenylsilyl)phenyl‐phosphine oxide) (TSPO1) (50:50). A low doping concentration of 1 wt% was used because fused planar ring compounds have the tendency to show aggregation in the solid state. The ultraviolet–visible (UV–vis) absorption spectra of the compounds (Figure S3a, Supporting Information) were similar, but the well‐resolved and sharp absorption bands observed in the BisICz and tBisICz emitters were red‐shifted in the tPBisICz emitter by the phenyl substituent, which extended the *π*‐conjugation. The absorption bands below 370 nm are assigned to the *π*–*π* ^∗^ transitions of the fused molecular structure,^[^ ^29^ ^]^ whereas those between 370 and 450 nm are attributed to the *n*–*π* ^∗^ transitions of the ICz part.^[^ ^30^ ^]^ Optical gaps were evaluated from absorption onsets of 2.84, 2.79, and 2.72 eV for BisICz, tBisICz, and tPBisICz, respectively.

**Figure 3:**
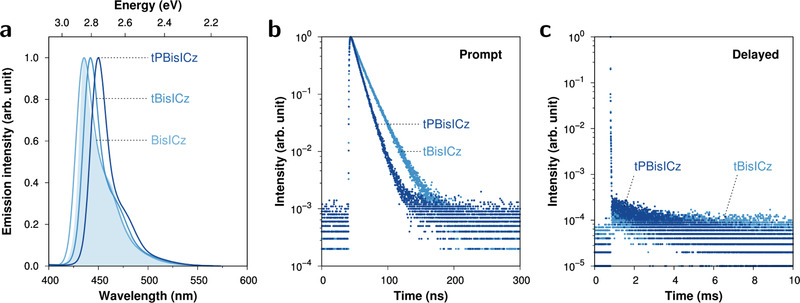
Photophysical properties of BisICz‐based emitters in 1 wt%‐doped mCP:TSPO1 film at 300 K. a) Steady‐state fluorescence spectra. Franck–Condon simulation of S_1_→S_0_ emission for BisICz (filled curve; red‐shifted by 6 nm). b,c) PL transient decay curves for (b) prompt and (c) delayed components. The corresponding plots for BisICz are given in Figure S6, Supporting Information, and no delayed component was observed.

The photoluminescence (PL) spectra of the emitters were collected from the thin films (**Figure** **3**a). The solution spectra are shown in Figure S3b–d, Supporting Information. The peak wavelengths of the film/solution PL spectra were 436/432, 442/442, and 450/448 nm for BisICz, tBisICz, and tPBisICz, respectively. The small red shift of the PL emission in the tBisICz and tPBisICz emitters was attributed to the electron‐donating substituents present in their structures; in particular, an ideal wavelength for ultrapure blue emission centered at 450 nm was obtained for tPBisICz. Owing to the rigid molecular structure based on the MR effect, the solid PL emission of BisICz was characterized by a small FWHM of 27 nm, whereas those of tBisICz and tPBisICz were characterized by an even smaller FWHM of 21 nm. Furthermore, BisICz and tBisICz showed similar Stokes shifts of 18 nm, whereas tPBisICz exhibited an even smaller Stokes shift of 13 nm. The small Stokes shift of the compounds points indeed to the rigidity of the coplanar molecular backbone structures due to MR effects. The Stokes shifts of the present emitters are significantly smaller than those of B/N‐based MR‐TADF emitters.^[^ ^7^ ^]^ The fluorescence spectra of the emitters in the mCP:TSPO1 film did not differ significantly from those in the dilute solution, which partly implies an efficient energy transfer from the host to our emitter. The small red shift of 6 nm can be ascribed to the stabilization of the emissive state by the host polarity under the amorphous film condition. The *E*_S_/*E*_T_/∆*E*_ST_ values were obtained as 2.89/2.58/0.31, 2.84/2.55/0.29, and 2.81/2.54/0.27 eV for BisICz, tBisICz and tPBisICz, respectively. The PL emission hardly changed in different solvents, implying that the singlet emission had hardly any CT character (Figure S4, Supporting Information).

Excellent PL quantum yield (PLQY) values of 95% and 91% were obtained for tBisICz and tPBisICz. A slightly lower value (81%) was obtained for BisICz presumably because of molecular aggregation. We observed short nanosecond decay for prompt fluorescence (Figure 3b) and significant decay for delayed fluorescence from tBisICz and tPBisICz, as indicated by the slow decay in the transient PL data (Figure 3c) and by the large delayed PLQY (Φ_DF_) values of 47% and 56%, respectively, which affirmed our design strategy for SVC‐TADF emitters. From recent report by Lian Duan's group, it was described that tBisICz did not show TADF properties.^[^ ^31^ ^]^ However, from temperature‐dependent transient PL, tBisICz, and tPBisICz showed increased delayed component by thermal activation, indicating that both emitters showed TADF phenomenon (Figure S5, Supporting Information). No delayed fluorescence was detected for BisICz (Figure S6, Supporting Information). From the prompt and delayed spectra measurement, tBisICz and tPBisICz showed delayed emission spectra, however, BisICz did not show any delayed emission (Figure S7, Supporting Information). The experimentally estimated *k*_RISC_ followed the inverse of the energy denominator ∆*E*_ST_ − ∆*E*_TT_, as expected in the on‐resonance SV model. We found that the radiative decay rates increased with the substitutions; the *k*_PF_ values were 5.4, 6.3, and 9.2 × 10*^7^* s^−1^ for BisICz, tBisICz, and tPBisICz, respectively; these values are in agreement with the theoretical predictions obtained for increasing oscillator strengths (Table S4, Supporting Information). Full details with fitting data are summarized in Table S5, Supporting Information. The thermal stability of the emitters was determined by thermogravimetric analysis (TGA). The decomposition temperature (*T*_d_) with a 5% weight loss for tPBisICz was estimated to be 522 °C by TGA (Figure S8, Supporting Information). Hence, tPBisICz shows higher thermal stability than BisICz (424 °C) and tBisICz (435 °C) because of the bulky di‐*tert*‐butylphenyl substituent and can be safely evaporated in a vacuum evaporator.

### 2.4 RISC Dynamics

To further elucidate the resonance condition that transformed a formerly pure fluorescent emitter BisICz into an SVC‐TADF emitter, we employed Fermi's golden rule in conjunction with the second‐order coupling (Equation (1)) for the quantitative analysis of *k*_RISC_:

(3) $$k_{\mathrm{RISC}}=\frac{2\pi }{\hbar }\sum_{vv^{\prime }}P_{v}\left(T\right){\left|H_{\mathrm{ST}}\right|}^{2}\delta \left(-\Delta E_{\mathrm{ST}}+E_{v}-E_{v^{\prime }}\right)$$

Here, the *δ*‐function imposes energy conservation on the T_1_ and S_1_ vibrational energies *E*_*ν*_ and *E*_*ν*′_ and the Boltzmann distribution *P*_*ν*_ describes the thermal population at temperature *T*. Because the DSO and SV terms in Equation (1) cannot mix (up to the second order) in the square of the coupling because of time‐reversal symmetry,^[^ ^32^ ^]^ *k*_RISC_ simply becomes a sum of *k*_DSO_ and *k*_SV_ from the respective squared terms. The calculated *k*_RISC_ values are listed in Table S2, Supporting Information, together with other related excited‐state properties.

Our computed *k*_RISC_ values of 1.47 and 0.27 × 10^3^ s^−1^ agree well quantitatively with the experimental values of 1.41 and 0.15 × 10^3^ s^−1^ for tPBisICz and tBisICz, respectively, measured under the thin film condition (Table S4, Supporting Information). Despite the similar ∆*E*_ST_ values for tPBisICz and tBisICz, the smaller energy denominator ∆*E*_ST_ − ∆*E*_TT_ in the former (48 cm^−1^) amplifies the electronic coupling dramatically and causes the rate constant of the former to be an order of magnitude greater than that of the latter. As anticipated from the combination of the small S_1_–T_1_ SOC and large ∆*E*_ST_, the RISC dynamics is completely governed by the SVC, that is, *k*_SV_/*k*_RISC_ ≃ 1.0 (Table S2, Supporting Information). Because of the larger mismatch between ∆*E*_ST_ and ∆*E*_TT_, a negligibly small *k*_RISC_ was obtained for BisICz. Detailed calculation methods of the rate constants are in the Supporting Information.

In all the emitters, the S_1_–T_2_ SOC, which plays a crucial role in the second‐order RISC, is a few times larger than the S_1_–T_1_ SOC. Specifically, the product of the S_1_–T_2_ SOC and T_1_–T_2_ NAC is a decisive factor, and therefore, we now focus on the mode‐wise deconvolution of *k*_RISC_ (Figure S9a, Supporting Information). We first project the non‐adiabatic components onto the vibrational modes. By visually inspecting the NAC vectors between the T_1_ and T_2_ states (Figure S9b, Supporting Information), one can expect that the modes making major contributions in the SVC‐RISC will be characterized as the stretched parts of the central benzene ring of the BisICz unit, which is distributed over four vibrational modes (Figure S9c, Supporting Information). The *k*_SV_ values arising from these modes account for ≈50% of the total *k*_RISC_ (Table S2, Supporting Information). Furthermore, another important factor for the SVC‐RISC dynamics is the geometrical displacement between the S_1_ and T_1_ states. We varied the displacement by a factor of two in our SV model and observed that the enhancement in *k*_SV_ is effectively quenched, indicating that the resonance in the electronic coupling becomes diluted, which is partly related to the poor vibrational overlap (Figure S9d,e, Supporting Information).

**Figure 4:**
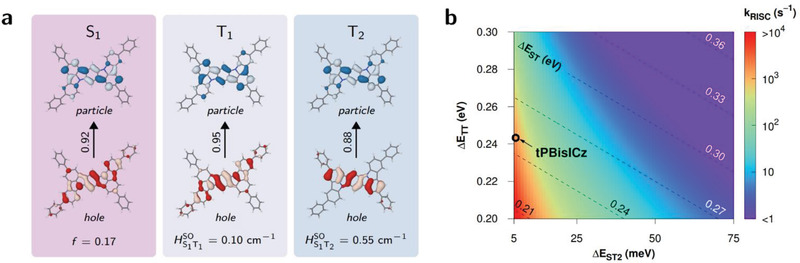
Molecular factors affecting *k*_RISC_. a) NTO pairs for excited states of tPBisICz. The weights are given beside the arrows. The oscillator strength and SOCs are given for the singlet and triplet states, respectively. b) *k*_RISC_ simulated as a function of ∆*E*_TT_ and ∆*E*_ST2_ (≡ |∆*E*_TT_ − ∆*E*_ST_|) for tPBisICz. The diagonal scale of ∆*E*_ST_ is also supplemented.

To understand how these key vibrations allow a strong non‐adiabatic mixing between the T_1_ and T_2_ states, we examined the natural transition orbitals (NTOs) of the involved states (**Figure** **4**a). The same particle wavefunction is rendered for the S_1_, T_1_, and T_2_ states. The hole wavefunctions of the S_1_ and T_1_ states are identical, whereas those of the T_1_ and T_2_ states differ, reflecting the NAC vectors to some extent. The hole wavefunction of T_2_ is localized in the Ph–Ph–Ph segment. This suggests that lowering the T_2_ state may not enhance the resonance observed here because this will start to perturb the T_1_ state with inevitable state mixing, leading to geometrical displacement in the central phenyl ring and consequent quenching of the SVC‐RISC process.

Figure 4b shows how SVC‐RISC enhancement varies with respect to the energy differences between the S_1_, T_1_, and T_2_ states using the molecular properties of tPBisICz. To gain a sizable enhancement in *k*_RISC_, simultaneous optimization should be carried out in all the three states such that ∆*E*_ST_ ≤ 0.26 eV and |∆*E*_ST_−∆*E*_TT_| ≤ 10 meV, which will ensure that *k*_SV_ ≈ 10^3^ s^−1^. This is important because the present mechanism provides a new pathway for triplet harvesting in rigid emitters with large ∆*E*_ST_.

### 2.5 OLED Device Characterization

**Figure 5:**
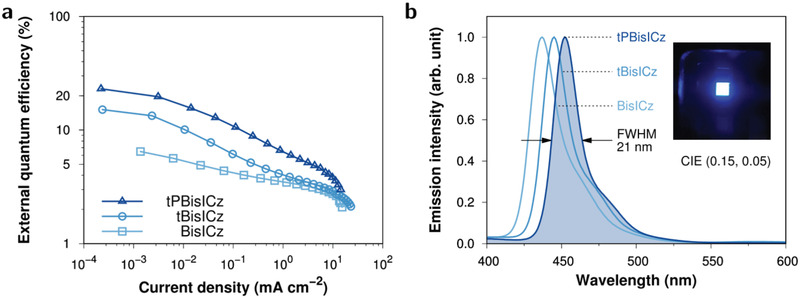
OLED performance of 1 wt%‐doped mCP:TSPO1 devices. a) EQE versus current density. b) Normalised EL spectra. The inset shows the EL of the device fabricated using tPBisICz (corresponding to the filled curve).

We demonstrated the potential of the ultrapure blue molecules as emitter materials in vacuum‐deposited OLEDs using the device structure in Figure S10, Supporting Information. The energy levels were measured by cyclic voltammetry, as detailed in Section S1.3.1, Supporting Information. The emissive layer consisted of the mCP:TSPO1 mixed host doped with ultrapure blue SVC‐TADF emitters at a concentration of 1 wt%. The resulting electroluminescence (EL) performances are presented in **Figure** **5** and Table S6, Supporting Information. The EL spectra were almost identical to the respective PL spectra (Figure 3a) both in terms of the peak maximum and FWHM, clearly indicating the same emissive origin of the S_1_ state regardless of the exciton generation mechanism. These rules out the contribution of any bimolecular species such as exciplexes to the EL emission. The EL spectra were not changed according to voltage (Figure S11, Supporting Information). Strikingly, the device with tPBisICz showed excellent efficiencies with a maximum EQE of 23.1% (which is the theoretical maximum), current efficiency of 13.5 cd A^−1^, and power efficiency of 13.3 lm W^−1^ (Figure S12, Supporting Information). EQE_max_ values followed the order of tPBisICz > tBisICz > BisICz. The EQE of the purely fluorescent BisICz device was low because triplet excitons could not be utilized for singlet emission. The impressive performance of the OLED with tPBisICz suggests the almost complete triplet‐to‐singlet population transfer, assisted by the resonant SVC. The lower *k*_RISC_ of tBisICz competes with other non‐radiative quenching processes such as triplet‐triplet annihilation or triplet‐polaron quenching, leading to the lower EQE. The high EQE of tPBisICz relative to that of tBisICz in spite of similar PLQY values was partly attributed to the high horizontal dipole orientation ratio, as shown in Figure S13, Supporting Information. As compared with the Lambertian distribution, the wide EL emission pattern from angle dependent EL measurement indicated that high EQE was not from microcavity or optical effect. (Figure S14, Supporting Information). Although the delayed fluorescence lifetime of the emitters is rather long in the sub‐millisecond range, the triplet excited states of the emitters can be effectively managed using a suitable device structure, like the one employed in this work. The delayed fluorescence was clearly observed in the transient EL measurement (Figure S15, Supporting Information).

In all the OLEDs, irrespective of the efficiencies, an ultrapure blue CIE*_y_* coordinate of ≤0.05 was achieved. The EL spectra of these SVC‐TADF‐emitter‐doped devices showed ultrapure emission with FWHM values as small as 21 nm. The color coordinates of the BisICz, tBisICz, and tPBisICz devices were (0.16, 0.04), (0.16, 0.05), and (0.15, 0.05), respectively. However, with the current device data, the efficiency roll‐off is rather severe because of the lack of a highly stable device structure to accommodate high‐energy triplet excitons and the annihilation of triplet excitons presumably due to a rather slow RISC conversion.

## 3 Conclusions

We designed ultrapure blue emitters with superior color purity based on an SV model, which was also used to elucidate the second‐order RISC dynamics mediated by high‐lying intermediary states and predominately driven by resonance conditions. Because of the energetic resonance between the involved states (S_1_ and T_2_) accompanied by large SOCs and non‐Born–Oppenheimer couplings, resonance was realized by electron‐donating substituents, thereby perturbing the frontier orbitals in a purely fluorescent BisICz molecule. The emitters (tBisICz and tPBisICz) showed a high PLQY (>90%), small Stokes shift (<20 nm), and narrow spectrum. The OLED fabricated using tPBisICz afforded a maximum EQE of 23.1%, a narrow FWHM of 21 nm, and an ultrapure blue color coordinate of (0.15, 0.05), which is very close to the industrial blue standard obtained without needing a narrow color filter. Such a high EL efficiency is mainly attributed to the kinetically slow but efficient RISC of the light emitting materials, which is, in turn, due to the resonance condition of the energy levels. The proposed principles offer a design strategy for large‐∆*E*_ST_ emitters, which were previously considered to be inoperable, by fine‐tuning the electronic energy levels.

## Conflict of Interest

The authors declare no conflict of interest.

## Author Contributions

V.V.P., H.L.L., and I.K. contributed equally to this work. I.K. and W.‐J.S. performed the theoretical calculations. V.V.P., H.L.L., and S.O.J. designed the molecules and assessed the synthetic feasibility of molecular candidates from the calculations. V.V.P. and H.L.L. synthesized the compounds and analyzed the chemical structures. S.O.J. and S.P. analyzed the spectroscopic data and electrochemical properties. K.H.L. and W.J.C. fabricated and tested the bottom‐emitting devices and characterized the electrical and optical properties of the thin films. J.H.K. performed the anisotropic horizontal orientation study. S.O.J. and J.Y.L. wrote the first version of the manuscript. All the authors contributed to the discussion, writing, and editing of the manuscript. H.C. organized the project and supervised the computational chemistry study. J.Y.L. supervised the device fabrication and the project.

## Supporting information

Supporting Information

## Data Availability

Research data are not shared.

## References

[1] M. A. Baldo , D. F. O'Brien , M. E. Thompson , S. R. Forrest , Phys. Rev. B 1999, 60, 14422.

[2] M. A. Baldo , D. F. O'Brien , Y. You , A. Shoustikov , S. Sibley , M. E. Thompson , S. R. Forrest , Nature 1998, 395, 151.

[3] H. Uoyama , K. Goushi , K. Shizu , H. Nomura , C. Adachi , Nature 2012, 492, 234. 23235877 10.1038/nature11687

[4] H. Kaji , H. Suzuki , T. Fukushima , K. Shizu , K. Suzuki , S. Kubo , T. Komino , H. Oiwa , F. Suzuki , A. Wakamiya , Y. Murata , C. Adachi , Nat. Commun. 2015, 6, 8476. 26477390 10.1038/ncomms9476 PMC4634127

[5] X.‐K. Chen , D. Kim , J.‐L. Brédas , Acc. Chem. Res. 2018, 51, 2215. 30141908 10.1021/acs.accounts.8b00174

[6] Z. Yang , Z. Mao , Z. Xie , Y. Zhang , S. Liu , J. Zhao , J. Xu , Z. Chi , M. P. Aldred , Chem. Soc. Rev. 2017, 46, 915. 28117864 10.1039/c6cs00368k

[7] T. Hatakeyama , K. Shiren , K. Nakajima , S. Nomura , S. Nakatsuka , K. Kinoshita , J. Ni , Y. Ono , T. Ikuta , Adv. Mater. 2016, 28, 2777. 26865384 10.1002/adma.201505491

[8] X. Liang , Z.‐P. Yan , H.‐B. Han , Z.‐G. Wu , Y.‐X. Zheng , H. Meng , J.‐L. Zuo , W. Huang , Angew. Chem., Int. Ed. 2018, 57, 11316. 10.1002/anie.201806323 29974588

[9] Y. Kondo , K. Yoshiura , S. Kitera , H. Nishi , S. Oda , H. Gotoh , Y. Sasada , M. Yanai , T. Hatakeyama , Nat. Photonics 2019, 13, 678.

[10] Y. Xu , C. Li , Z. Li , Q. Wang , X. Cai , J. Wei , Y. Wang , Angew. Chem., Int. Ed. 2020, 59, 17442. 10.1002/anie.202007210 32533603

[11] S. M. Suresh , E. Duda , D. Hall , Z. Yao , S. Bagnich , A. M. Z. Slawin , H. Bässler , D. Beljonne , M. Buck , Y. Olivier , A. Köhler , E. Zysman‐Colman , J. Am. Chem. Soc. 2020, 142, 6588. 32134259 10.1021/jacs.9b13704

[12] J. U. Kim , I. S. Park , C.‐Y. Chan , M. Tanaka , Y. Tsuchiya , H. Nakanotani , C. Adachi , Nat. Commun. 2020, 11, 1765. 32286281 10.1038/s41467-020-15558-5 PMC7156453

[13] D. H. Ahn , S. W. Kim , H. Lee , I. J. Ko , D. Karthik , J. Y. Lee , J. H. Kwon , Nat. Photonics 2019, 13, 540.

[14] X. Li , Y.‐Z. Shi , K. Wang , M. Zhang , C.‐J. Zheng , D.‐M. Sun , G.‐L. Dai , X.‐C. Fan , D.‐Q. Wang , W. Liu , Y.‐Q. Li , J. Yu , X.‐M. Ou , C. Adachi , X.‐H. Zhang , ACS Appl. Mater. Interfaces 2019, 11, 13472. 30892014 10.1021/acsami.8b19635

[15] Y. Yuan , X. Tang , X.‐Y. Du , Y. Hu , Y.‐J. Yu , Z.‐Q. Jiang , L.‐S. Liao , S.‐T. Lee , Adv. Opt. Mater. 2019, 7, 1801536.

[16] S. M. Suresh , D. Hall , D. Beljonne , Y. Olivier , E. Zysman‐Colman , Adv. Funct. Mater. 2020, 30, 1908677.

[17] *International Telecommunication Union*, Recommendation BT.2020 (08/2012), https://www.itu.int/rec/R‐REC‐BT.2020‐0‐201208‐S/en (accessed: January 2020).

[18] A. Khan , X. Tang , C. Zhong , Q. Wang , S.‐Y. Yang , F.‐C. Kong , S. Yuan , A. S. D. Sandanayaka , C. Adachi , Z.‐Q. Jiang , L.‐S. Liao , Adv. Funct. Mater. 2021, 31, 2009488.

[19] H. L. Lee , W. J. Chung , J. Y. Lee , Small 2020, 16, 1907569.

[20] J. Gibson , A. P. Monkman , T. J. Penfold , ChemPhysChem 2016, 17, 2956. 27338655 10.1002/cphc.201600662 PMC5096030

[21] M. K. Etherington , J. Gibson , H. F. Higginbotham , T. J. Penfold , A. P. Monkman , Nat. Commun. 2016, 7, 13680. 27901046 10.1038/ncomms13680 PMC5141373

[22] A. Pershin , D. Hall , V. Lemaur , J.‐C. Sancho‐Garcia , L. Muccioli , E. Zysman‐Colman , D. Beljonne , Y. Olivier , Nat. Commun. 2019, 10, 597. 30723203 10.1038/s41467-019-08495-5 PMC6363735

[23] P. de Silva , J. Phys. Chem. Lett. 2019, 10, 5674. 31483656 10.1021/acs.jpclett.9b02333

[24] Y. Zhang , D. Zhang , T. Tsuboi , Y. Qiu , L. Duan , Sci. China: Chem. 2019, 62, 393.

[25] B. R. Henry , W. Siebrand , J. Chem. Phys. 1971, 54, 1072.

[26] T. J. Penfold , E. Gindensperger , C. Daniel , C. M. Marian , Chem. Rev. 2018, 118, 6975. 29558159 10.1021/acs.chemrev.7b00617

[27] M. A. El‐Sayed , J. Chem. Phys. 1963, 38, 2834.

[28] I. Kim , K. H. Cho , S. O. Jeon , W.‐J. Son , D. Kim , Y. M. Rhee , I. Jang , H. Choi , D. S. Kim , JACS Au 2021, 10.26434/chemrxiv.12413417.v1. PMC8395647 34467345

[29] H. Li , Y. Wang , K. Yuan , Y. Tao , R. Chen , C. Zheng , X. Zhou , J. Li , W. Huang , Chem. Commun. 2014, 50, 15760. 10.1039/c4cc06636g 25370829

[30] P. Kautny , C. Zhao , D. Schopf , B. Stöger , E. Horkel , J. Chen , D. Ma , J. Foröhlich , D. Lumpi , J. Mater. Chem. C 2017, 5, 1997.

[31] J. Wei , C. Zhang , D. Zhang , Y. Zhang , Z. Liu , Z. Li , G. Yu , L. Duan , Angew. Chem., Int. Ed. 2021, 60, 12269. 10.1002/anie.202017328 33742743

[32] I. Kim , S. O. Jeon , D. Jeong , H. Choi , W.‐J. Son , D. Kim , Y. M. Rhee , H. S. Lee , J. Chem. Theory Comput. 2020, 16, 621. 31841330 10.1021/acs.jctc.9b01014

