# Supplementary material for: Purely Spin‐Vibronic Coupling Assisted Triplet to Singlet Up‐Conversion for Real Deep Blue Organic Light‐Emitting Diodes with Over 20% Efficiency and y Color Coordinate of 0.05
Source: Adv Sci (Weinh). 2021 Aug 13;8(20):2101137. doi: 10.1002/advs.202101137 (PMC8529479; doi:10.1002/advs.202101137)
Supplement: Supplementary file 1 — Supporting Information [file ADVS-8-2101137-s001.pdf]

© 2021 Wiley-VCH GmbH

ADVANCED SCIENCE
Open Access

Supporting Information

for *Adv. Sci.*, DOI: 10.1002/advs.202101137

Purely spin-vibronic coupling assisted triplet to singlet up-conversion for real deep blue organic light-emitting diodes with over 20% efficiency and y color coordinate of 0.05

Vilas Venunath Patil, Ha Lim Lee,* Inkoo Kim,**
Kyung Hyung Lee

Supplementary Information

Purely spin-vibronic coupling assisted triplet to singlet up-conversion for real deep blue organic light-emitting diodes with over 20% efficiency and y color coordinate of 0.05

Vilas Venunath Patil,[1,*] Ha Lim Lee,[1,*] Inkoo Kim,[2,*] Kyung Hyung Lee,[1]

Won Jae Chung,[1] Joonghyuk Kim,[3] Sangho Park,[3] Hyeonho Choi,[3]

Won-Joon Son,[2] Soon Ok Jeon,[3,†] and Jun Yeob Lee[1,‡]

[1]School of Chemical Engineering, Sungkyunkwan University, Suwon 16419, Korea.

[2]Data and Information Technology Center, Samsung Electronics, Hwaseong 18448, Korea.

[3]Samsung Advanced Institute of Technology, Samsung Electronics, Suwon 16678,Korea.

Email: so.jeon@samsung.com, leej17@skku.edu

Table of contents

1. Methods **S3**

1.1 Synthesis **S3**

1.1.1 General procedures. S3

1.1.2 Synthesis of ICz-based materials S4

1.2 Calculation **S8**

1.3 Optical properties and OLED devices **S11**

1.3.1 Optical characterisation methods S11

1.3.2 Photophysical properties of BisCz, tBisICz, and tPBIsICz S13

1.3.3 Characterisation of thermal analyses S18

1.3.4 RISC dynamics calculations S19

1.4 Device fabrication and performances **S20**

Supplementary Tables **S27**

Supplementary References **S30**

1.Methods

1.1 Synthesis

1.1.1. General procedures

All the reagents and solvents were procured from commercial sources and used without further purification. Chromatographic separations were performed using silica gel (200–300 nm). The purification of all the compounds was carried out by column chromatography, and for the final products, further purification was performed by a temperature gradient vacuum sublimation process. Each intermediatewas confirmed by high-resolution mass spectrometry (JMS-700, JEOL).The final products were confirmed by ^{1}H NMR spectra,which were recorded on a Bruker ASCEND 500 device at 500 MHz using CD_2Cl_2 and C_6D_6.^{13}C NMR spectrumoftBisICz was recorded on thesameinstrument using $CDCl_3$butin case of BisICz and tPBisICz, ^{13}C NMR spectra could not be obtained because of poor solubility. BothBisICzandtPBisICzidentified the molecular weights of compounds after the sublimation process using highresolution liquid chromatograph mass spectrometer–ion trap–time-of-flight (LCMS-IT-TOF) equipment. The measurement conditions of the LCMS-IT-TOF(Shimadzu) equipment were set as follows: acetonitrile and water as mobile phase solvents, a 1.0 mL/min flow rate, and tetrahydrofuran as a sampling solvent. Atmosphericpressurechemicalionization(APCI)was used for obtaininganionizedsource.Kinetex 2.6 μm Biphenyl 100A, 4.6 × 100 mm (Phenomenex) was used as the column. Elemental analysis (EA) was performed usingan organic elemental analyser (vario EL cube, Elementar).

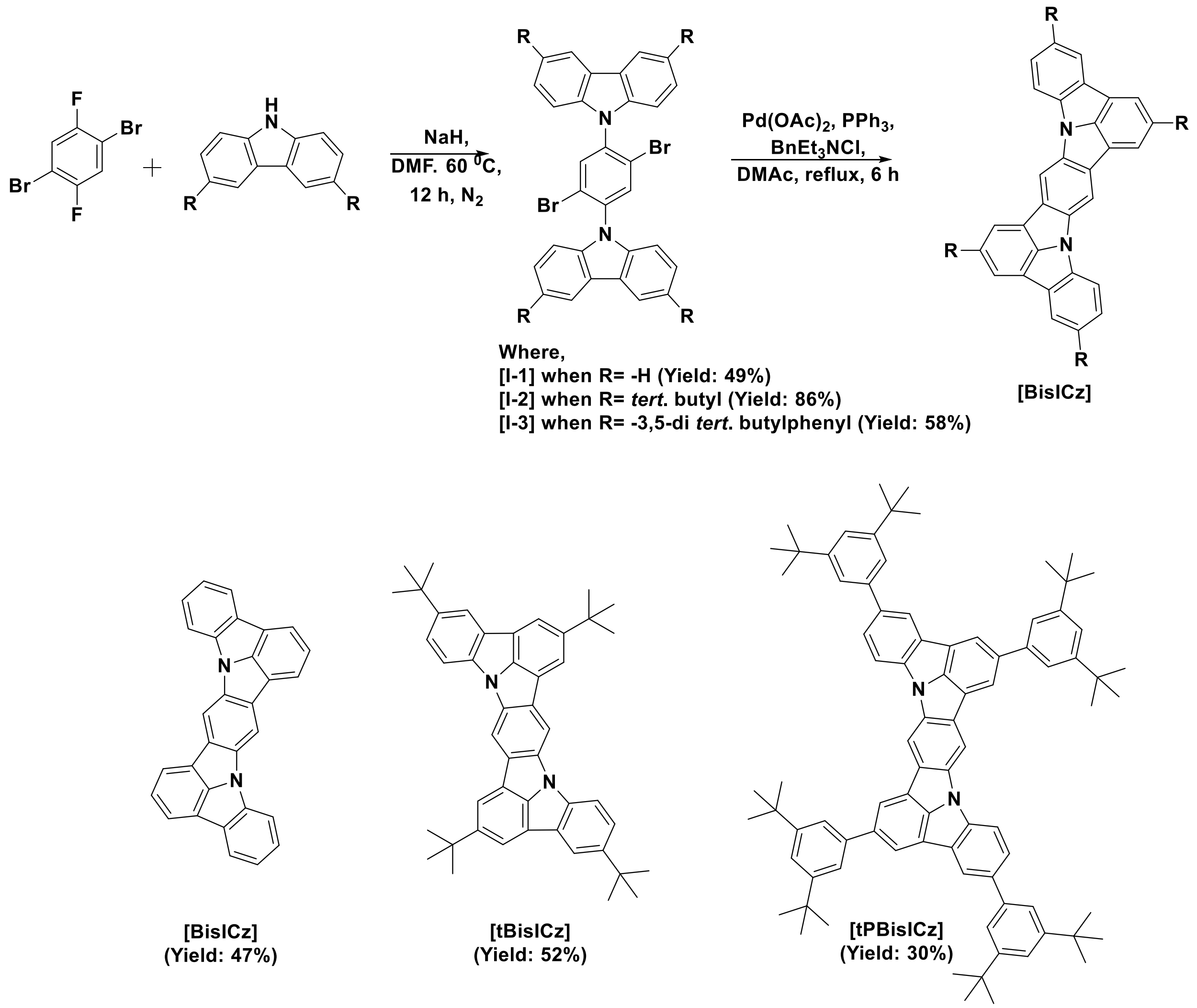

Supplementary Scheme 1. Synthetic scheme of BisICz-based materials

1.1.2 Synthesis of ICz-based materials

9,9′-(2,5-Dibromo-1,4-phenylene)bis(9*H*-carbazole) (I-1)

In a 250 mL round-bottomed flask, a mixture of sodium hydride (NaH, 1.2 g, 29.9 mmol, 60% dispersion in mineral oil), 9*H*-carbazole (5.00 g, 29.9 mmol), and 1,4-dibromo-2,5-difluorobenzene (6.10 g, 22.4 mmol) was stirred in 50 mL of anhydrous *N*,*N*-dimethyl formamide (DMF) for 30 min at 25 °C under a nitrogen atmosphere. The mixture was heated to 60 °C for 12 h. After the completion of the

reaction, the reaction mass was cooled to 25 °C and diluted with 200 mL of water. The solid obtained was filtered and washed with excess methanol. The crude product obtained was dried at 65 °C in a vacuum oven overnight to give 9,9′-(2,5-dibromo-1,4-phenylene)bis(9*H*-carbazole) **(I-1)** as a white solid. Yield (4.15 g, 49%).^{1}H NMR (500 MHz, $CDCl_3$) δ 8.18 (d, J = 7.8 Hz, 4H), 8.00 (s, 2H), 7.50 (ddd, J = 8.2, 7.3, 1.1 Hz, 4H), 7.38 – 7.35 (m, 4H), 7.26 (s, 2H), 7.24 (s, 2H). HRMS (FAB) m/z, found: 568.14 [$(M+H)^+$]. Calculated for $C_{30}H_{18}Br_2N_2$: 566.30.

Indolo[3,2,1-*jk*]indolo[1′,2′,3′:1,7]indolo[3,2-*b*]carbazole (BisICz)

A three-neck round-bottomed flask was charged with 9,9′-(2,5-dibromo-1,4-phenylene)bis(9*H*-carbazole) **(I-1)** (1.50 g, 2.65 mmol), triphenylphosphine (PPh_3, 0.40 g, 1.59 mmol), potassium carbonate (K_2CO_3, 2.90 g, 21.19 mmol), palladium (II) acetate ($Pd(OAc)_2$, 0.15 g, 0.66 mmol, 0.25), and benzyltriethylammonium chloride ($BnEt_3NCl$, 1.20 g, 5.30 mmol, 2 equiv.) in *N*,*N*-dimethyl acetamide (DMAc, 30 mL). The reaction mixture was refluxed for 6 h under a nitrogen atmosphere. After the completion of the reaction, the reaction mixture was cooled to 25 °C and 50 mL of methanol was added to the slurry. The solid was filtered and washed with excess methanol. The crude product was purified by sublimation to give **BisICz** as a bright yellow solid. Yield (0.5 g, 47%). ^{1}H NMR (500 MHz, CD_2Cl_2) δ 8.73 (s, 2H), 8.26 (d, J = 7.3 Hz, 2H), 8.23 (d, J = 7.5 Hz, 2H), 8.16 (d, J = 7.4 Hz, 2H), 8.15 (d, J = 7.3 Hz, 2H), 7.70 (t, J = 7.4 Hz, 2H), 7.68 (t, J = 7.3 Hz, 2H), 7.45 (t, J = 7.5 Hz, 2H). LCMS (APCI) m/z,found: 404.1313 [$(M)^+$]. Calculated for $C_{30}H_{16}N_2$: 404.1314. EA (%): calculated for CHN: C, 89.09; H,3.99; N, 6.93. Found: C,88.83; H,3.96; N,7.09.

9,9′-(2,5-Dibromo-1,4-phenylene)bis(3,6-di-*tert*-butyl-9*H*-carbazole) (I-2)

The intermediate **I-2** was synthesised using the same procedure used for **I-1**. 9*H*-Carbazole was replaced with 3,6-di-*tert*-butyl-9*H*-carbazole (3.00 g, 10.73 mmol) under the same reaction conditions using 1,4-dibromo-2,5-difluorobenzene (2.20 g, 8.05 mmol), NaH (0.50 g, 10.73 mmol), and DMF (30 mL). The final product **I-2** was obtained as a white solid. Yield (3.66 g, 86%). ^{1}H NMR (500 MHz, $CDCl_3$) δ 8.17 (d, J = 1.6 Hz, 4H), 7.93 (s, 2H), 7.53 (dd, J = 8.6, 1.7 Hz, 4H), 7.16 (d, J = 8.6 Hz, 4H), 1.49 (s, 36H). HRMS (FAB) m/z, found: 791.80 [$(M+H)^+$]. Calculated for $C_{46}H_{50}Br_2N_2$: 790.73.

2,5,11,14-Tetrakis(1,1-dimethylethyl)indolo[3,2,1-*jk*]indolo[1′,2′,3′:1,7]indolo[3,2-*b*]carbazole (tBisICz)

tBisICz was synthesised using the same procedure used for **BisICz**. 9,9′-(2,5-Dibromo-1,4-phenylene)bis(3,6-di-*tert*-butyl-9*H*-carbazole) **(I-2)** (1.50 g, 1.89 mmol), PPh_3 (0.30 g, 1.14 mmol), K_2CO_3 (2.10 g, 15.18 mmol), $Pd(OAc)_2$ (0.11 g, 0.47 mmol), and $BnEt_3NCl$ (0.90 g, 3.79 mmol) in DMAc (30 mL) were used for the reaction. **tBisICz** was obtained as a yellow solid. Yield (0.62 g, 52%). ^{1}H NMR (500 MHz, C_6D_6) δ 8.61 (s, 2H), 8.43 (s, 2H), 8.35 (s, 4H), 7.81 (d, J = 8.4 Hz, 2H), 7.64 (d, J = 8.4 Hz, 2H), 1.63 (s, 18 H), 1.47 (s, 18H). ^{13}C NMR (126 MHz, $CDCl_3$) δ 146.72, 144.62, 144.15, 137.46, 135.26, 130.07, 129.52, 124.22, 119.95, 118.33 (d, *J*= 12.4 Hz), 117.02, 116.55, 111.64, 106.37, 36.26, 35.15, 33.14, 32.19.LCMS (APCI) m/z,found: 628.3817 [$(M)^+$]. Calculated for $C_{46}H_{48}N_2$: 628.3813. EA (%): calculated for CHN: C,87.85; H,7.69; N,4.45. Found: C,87.67; H,7.69; N,4.67.

9,9′-(2,5-Dibromo-1,4-phenylene)bis(3,6-bis(3,5-di-*tert*-butylphenyl)-9*H*-carbazole) (I-3)

The intermediate **I-3** was synthesised using the same procedure used for **I-1**. 9*H*-Carbazole was replaced with 3,6-bis(3,5-di-*tert*-butylphenyl)-9*H*-carbazole (1.00 g, 1.84 mmol) under the same reaction conditions using 1,4-dibromo-2,5-difluorobenzene (0.37 g, 1.38 mmol), NaH (0.09 g, 1.84 mmol), and DMF (15 mL). The final product **I-3** was obtained as a white solid. Yield (1.40 g, 58%).^{1}H NMR (500

MHz, $CDCl_3$) δ 8.41 (d, J = 1.5 Hz, 4H), 8.10 (s, 2H), 7.75 (dd, J = 8.4, 1.7 Hz, 4H), 7.56 (d, J = 1.7 Hz, 8H), 7.48 (s, 4H), 7.36 (d, J = 8.5 Hz, 4H), 1.44 (s, 72H). HRMS (FAB) m/z, found: 1320.60 [$(M+H)^+$]. Calculated for $C_{86}H_{98}Br_2N_2$: 1319.52.

2,5,11,14-Tetrakis(3,5-di-*tert*-butylphenyl)-indolo[3,2,1-*jk*]indolo[1′,2′,3′:1,7]indolo[3,2-*b*]carbazole (tPBisICz)

tPBisICz was synthesised using the same procedure used for **BisICz**. 9,9′-(2,5-Dibromo-1,4-phenylene)bis(3,6-bis(3,5-di-*tert*-butylphenyl)-9*H*-carbazole) **I-4** (1.00 g, 0.76 mmol), PPh_3 (0.12 g, 0.46 mmol), K_2CO_3 (0.84 g, 6.1 mmol), $Pd(OAc)_2$ (0.05 g, 0.21 mmol), and $BnEt_3NCl$ (0.35 g, 1.52 mmol) in DMAc (20 mL) were used for the reaction. **tPBisICz** was obtained as a yellow solid. Yield (0.26 g, 30%). ^1H NMR (500 MHz, $CDCl_3$) δ 8.75 (s, 2H), 8.44 (s, 2H), 8.43 (s, 2H), 8.37 (s, 2H), 8.22 (d, J = 8.3 Hz, 2H), 7.88 (d, J = 8.3 Hz, 2H), 7.68 (d, J = 1.5 Hz, 4H), 7.61(d, J = 1.5 Hz, 4H), 7.52 (s, 2H), 7.50 (s, 2H), 1.49 (s, 36H), 1.46 (s, 36H). EA (%): calculated for $C_{86}H_{96}N_2$; C,89.22; H,8.36; N,2.42. Found: C,89.40; H,8.16; N,2.74.

1.2. Calculations

Electronic structure calculations were performed with TURBOMOLE 7.3[S1] and Q-Chem 5.3[S2] program packages. With the original spin-component scaling parameters[S3], the excited state geometries were optimized by the strict variant of the second-order algebraic diagrammatic construction (SCS-ADC(2)) method[S4], whereas the ground state geometries were obtained from the second-order Møller-Plesset perturbation theory (SCS-MP2). The resolution-of-the-identity approximation for the electron repulsion integrals were used in treating correlations. For normal mode calculations, numerical Hessians were employed with a frequency scaling factor of 0.9631[S5]. The vibrational analysis proved that there is no imaginary frequency, indicating an energy minimum.

At the S_1 geometries, time-dependent DFT calculations under the Tamm−Dancoff approximation was adopted with the gap-tuned ω^*B97M-V functional[S6,S7] for the evaluation of spin−orbit coupling, using the one-electron Breit−Pauli operator, and first-order nonadiabatic coupling vectors between the excited-states.Solvent effects were treated with the polarizable continuum model in the integral equation formalism (IEF-PCM)[S8]. The amorphous molecular solid film conditionwas imitated by the parameters of ε = 2.84 and n = 1.7, derived from a bluehost material of mCP. The def2-SVP basis set was used for all calculations[S9].

Under the harmonic approximation,the analytical solution for rate equation (3) can be obtained in the framework of a correlationfunction[S10–S13] in conjunction with Duschinsky transformations[S14]. An in-house code[S13] was used for the rate calculations with the time integration interval of [−6553.6:6553.6] fs with a time stepof 0.1 fs.

Natural transition orbital (NTO) analyseswere postprocessed by TheoDORE 2.0 program[S15].

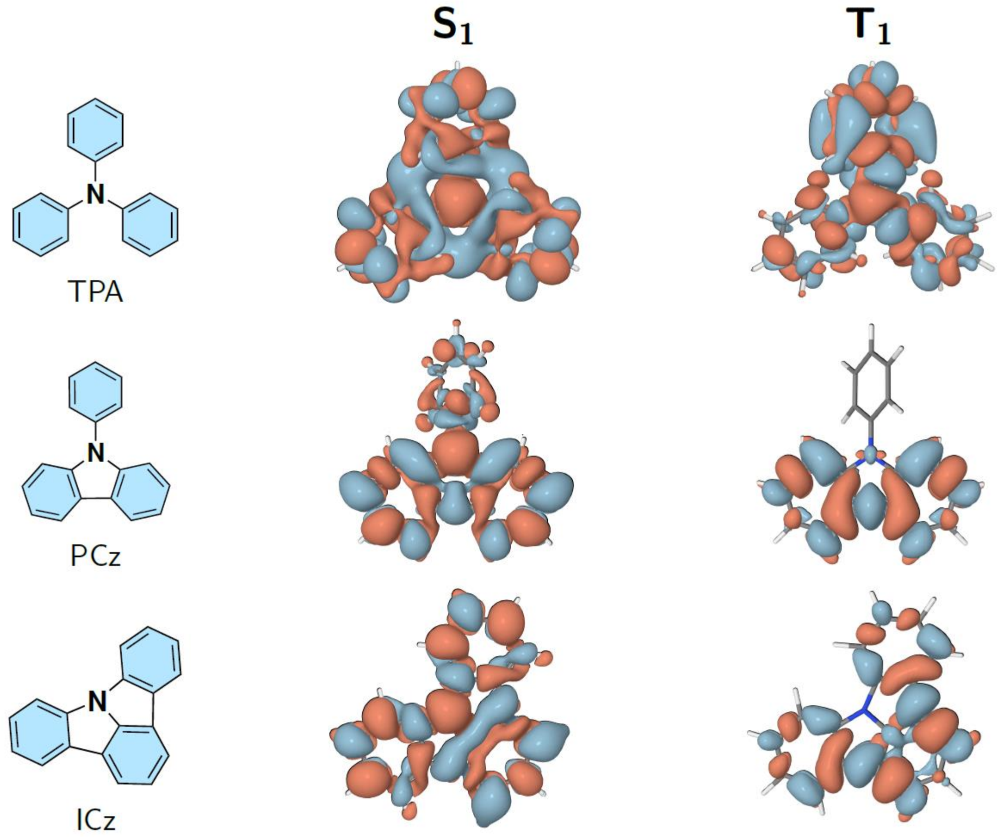

Supplementary Figure 1. Difference density plot of excited states for TPA, PCZ, and ICz.

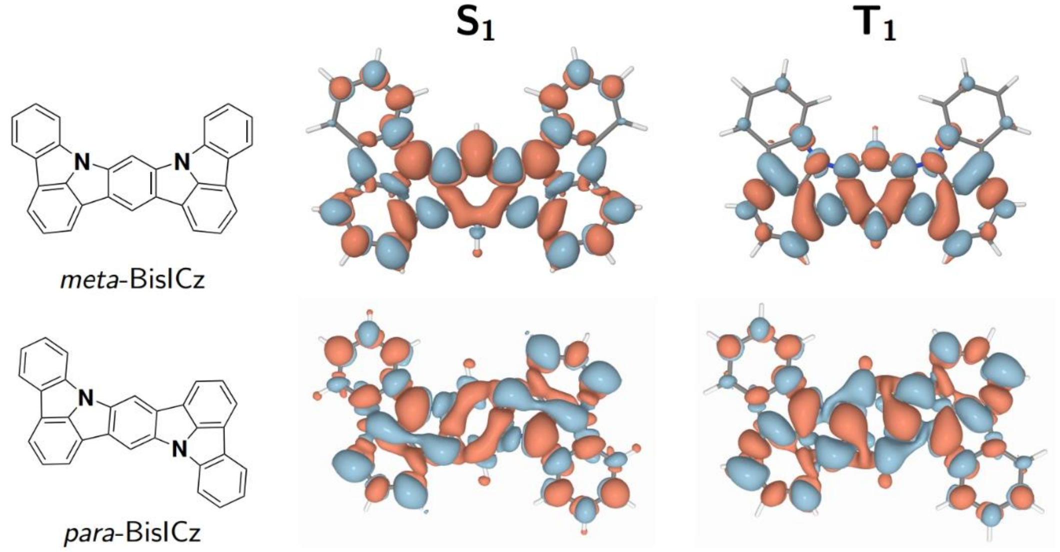

Supplementary Figure 2. Difference density plot of excited states for *meta*- and *para*-oriented BisICz.

1.3 Optical properties and OLED devices

1.3.1 Optical characterisationmethods

The ultraviolet-visible (UV-Vis) absorption and solution photoluminescence (PL) emission spectra of the materials were obtained from a dilute dichloromethane (DCM) solution (1 × 10^{-5} M), whereas the solid PL spectra were obtained from thin films prepared by vacuum evaporation. Triplet energy values of the ICz-based materials were obtained from the photoluminescence spectra at 77 K using liquid nitrogen with a 1 ms delay. The energy levels were measured by cyclic voltammetry. Each material was dissolved in anhydrous acetonitrile with 0.1 M tetrabutylammonium perchlorate as the electrolyte to measure the oxidation from which the HOMO energy level was estimated. A glassy carbon tube was used as the working electrode, a platinum wire was used as the counter electrode, and saturated Ag/AgCl was used the reference electrode. Ferrocene was used as the standard reference. All the solutions were purged with nitrogen for 10 min before each experiment. The LUMO energy was calculated from the UV-Vis gap. Thermogravimetric-differential thermal analysis (TG-DTA) was performed using a thermogravimetry/differential thermal analyser (Exstar 6000, TG/DTA6100, Seiko) under flowing nitrogen at a heating rate of 10 °C/min from room temperature to 600 °C to detect the decomposition temperature (T_d, corresponding to a 5% weight loss) of each TADF material.

The PL quantum yield and transient PL curves in the solid film were detected using a Quantaurus-QY system (Hamamatsu, C11347-11) and Quantaurus-Tau system (Hamamatsu, C11367-31). The electrical and optical characterisations of the devices were carried out using a Keithley 2400 Source Meter and CS 2000 spectroradiometer, respectively.

The rate constants were determined by using followed equation, reported in [S17].

$$k_{PF} = \frac{\mathbf{1}}{\tau_{PF}}$$

$$k_{DF} = \frac{\mathbf{1}}{\tau_{DF}}$$

$$k_{ISC} = (1 - \phi_F)k_p$$

$$\frac{k_{RISC} = \frac{k_p k_d}{k_{ISC}}\, \emptyset_{DF}}{\emptyset_{PF}}$$

Where, k_{PF} is prompt fluorescence rate constant, k_{DF} is delayed fluorescence rate constant, τ_{PF} is prompt fluorescence lifetime, τ_{DF} is delayed fluorescence lifetime, k_{ISC} is intersystem crossing (ISC) rate constant, ϕ_{PF} is prompt efficiency, k_{RISC} is reverse ISC (RISC) rate constant and ϕ_{DF} is delayed fluorescenceefficiency. Above all equation involved assumption that non-radiative singlet exciton is zero.

1.3.2 Photophysical properties of BisICz, tBisICz, and tPBisICz

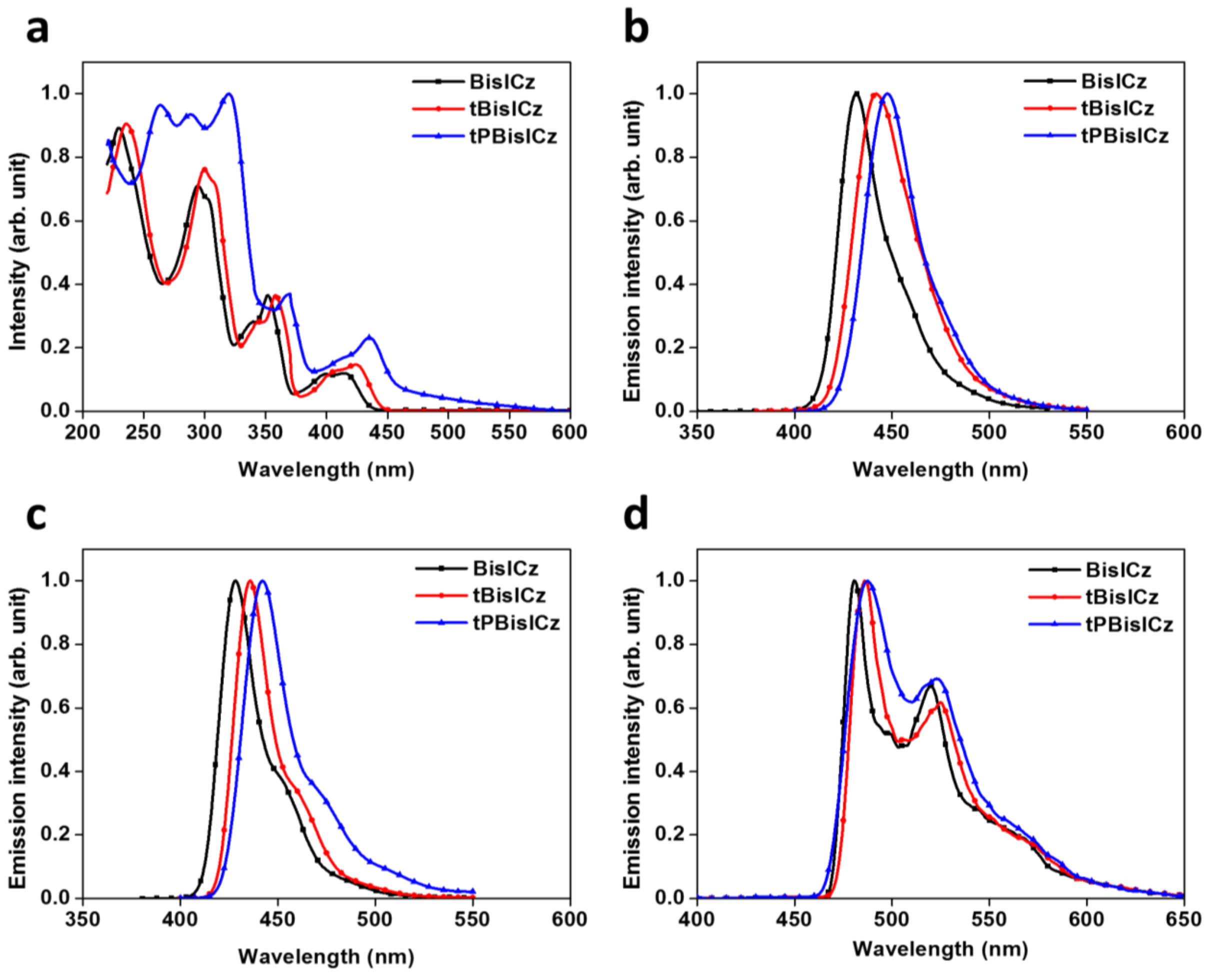

Supplementary Figure 3. Absorbance and photoluminescence spectra. a, UV-Vis, **b**,solution PL, **c**,low-temperature fluorescence at 77 K, and **d**,low-temperature phosphorescence at 77 K after delay time of 1 ms.

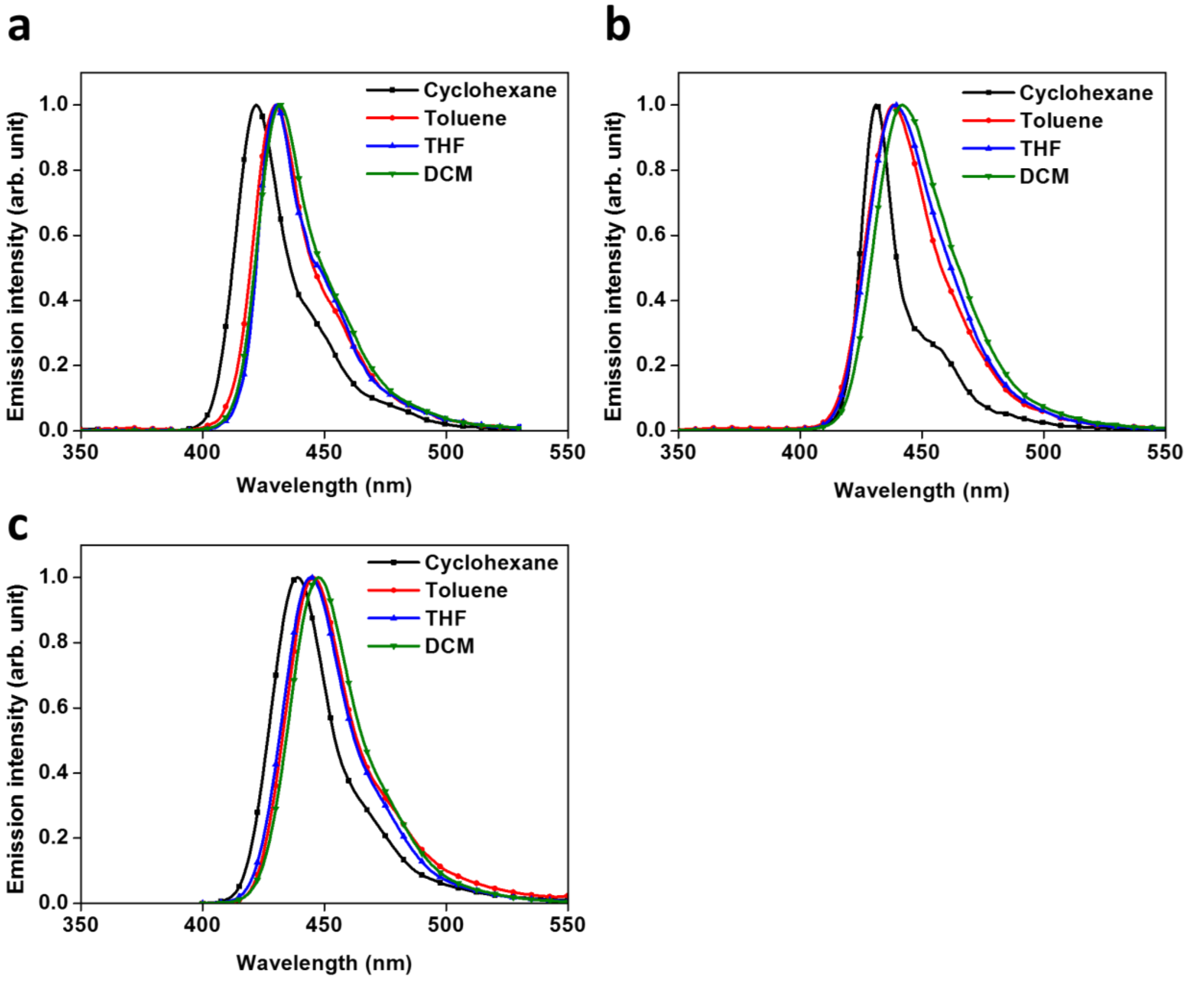

Supplementary Figure 4. Characterisation of emission in different solvents. **a**, **BisICz**, **b**, **tBisICz**, and **c**, **tPBisICz**.

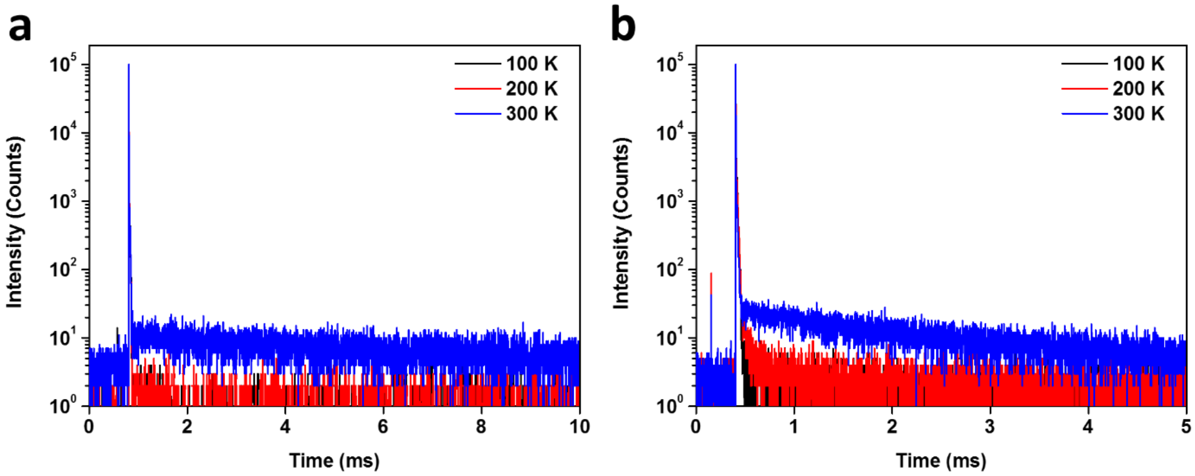

Supplementary Figure 5. Temperature-dependent transient PL curves. a, **tBisICz** and **b**, **tPBisICz**

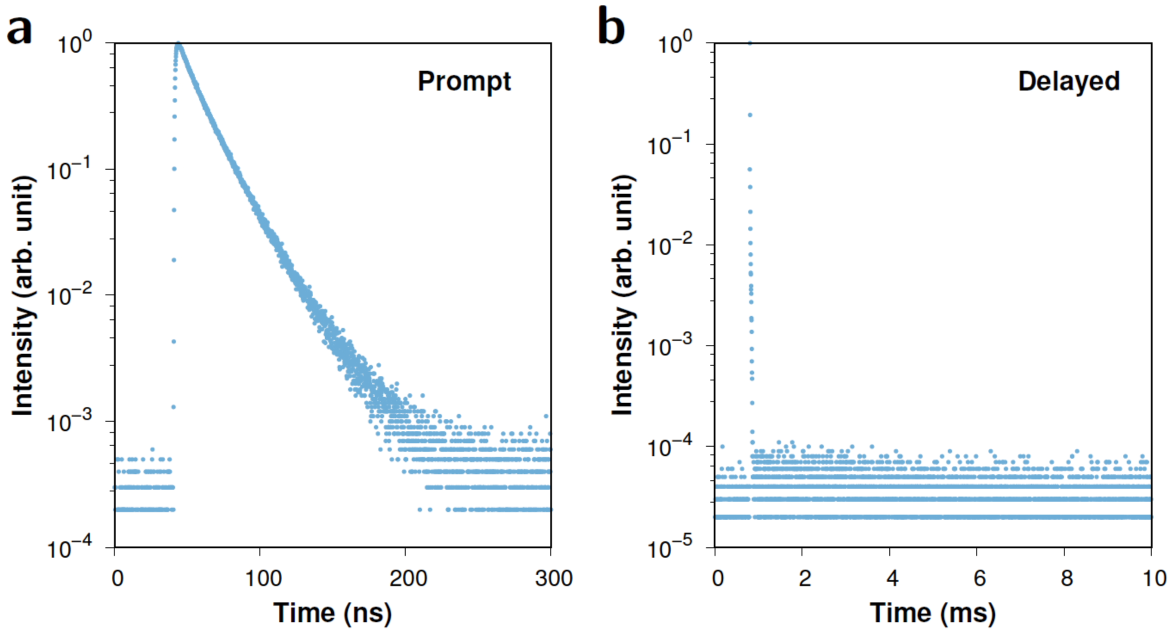

Supplementary Figure 6. Transient PL curves of BisICz. a,prompt and **b**,delayed components

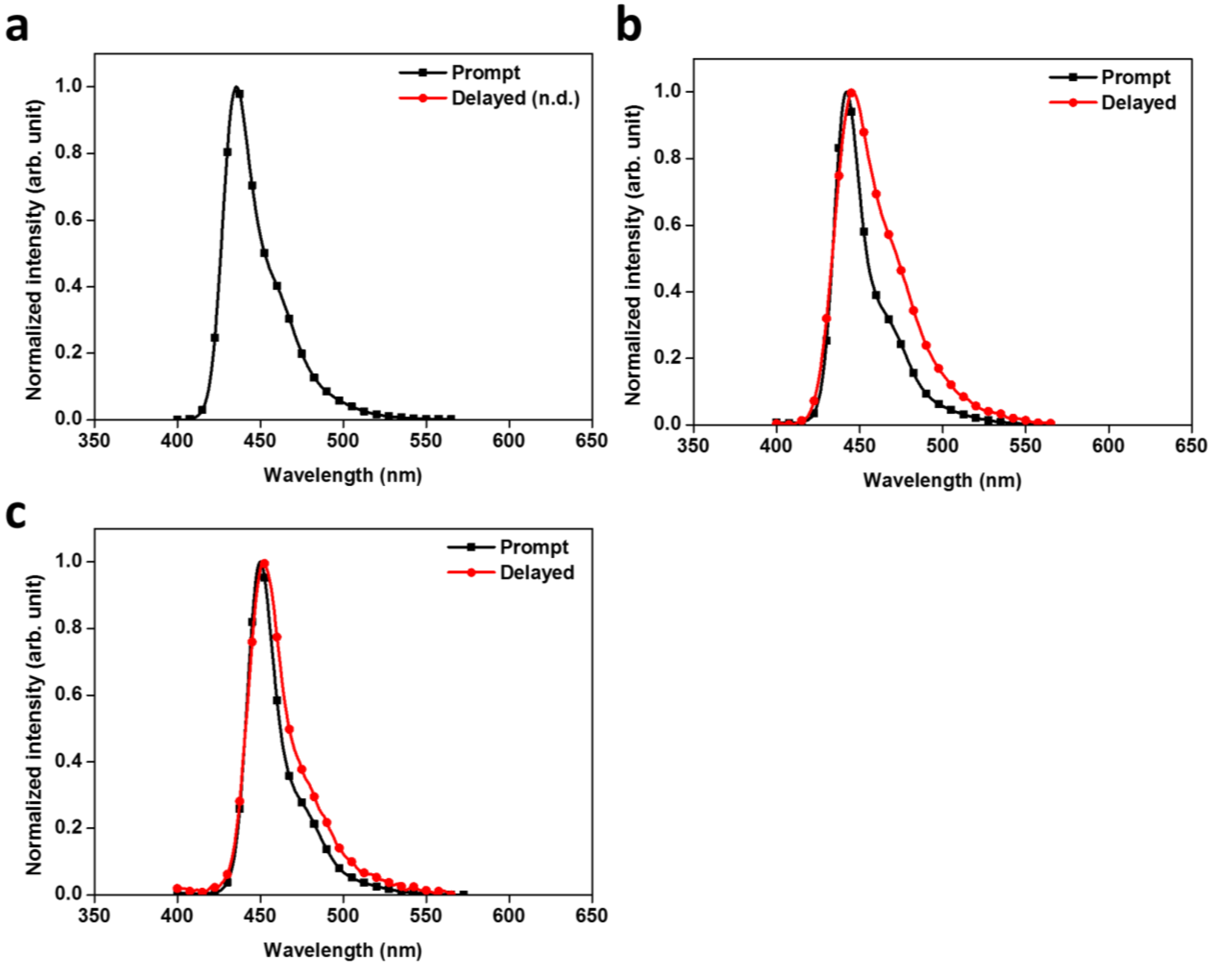

Supplementary Figure 7. Prompt and delayed fluorescence spectra. a, **BisICz, b**, **tBisICz** and **c**, **tPBisICz**

1.3.3 Characterisation of thermal analyses

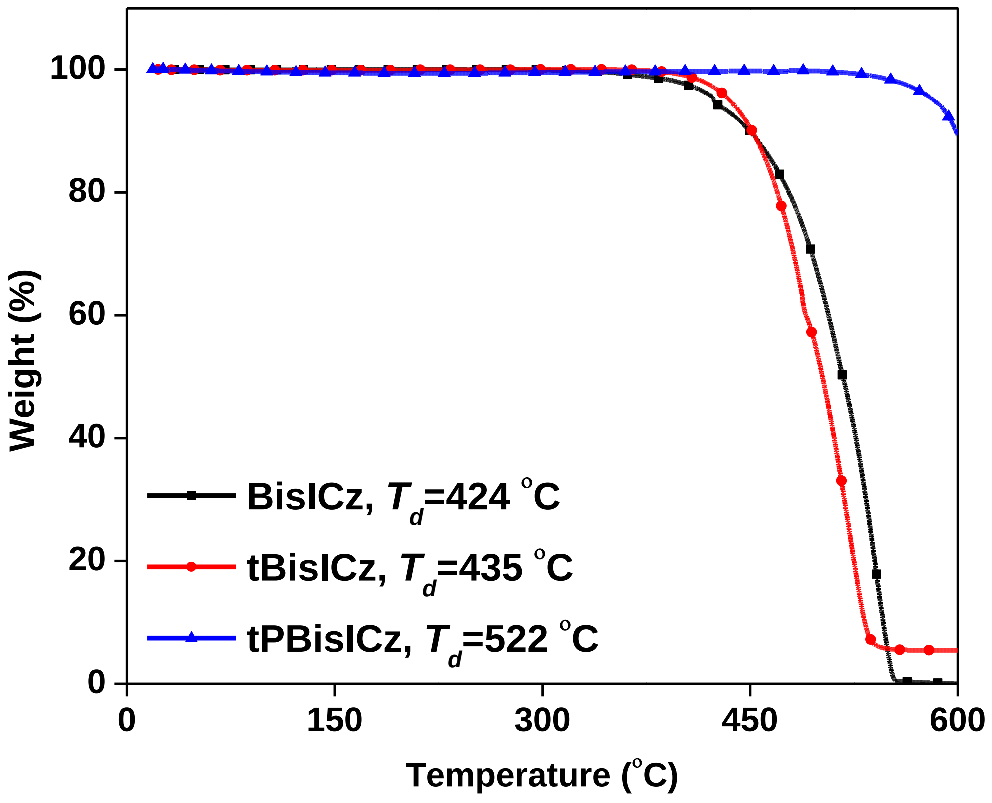

Supplementary Figure 8.Decomposition temperature (T_d) with 5% weight loss for BisICz, tBisICz, and tPBisICz.

1.3.4. RISC dynamicscalculations

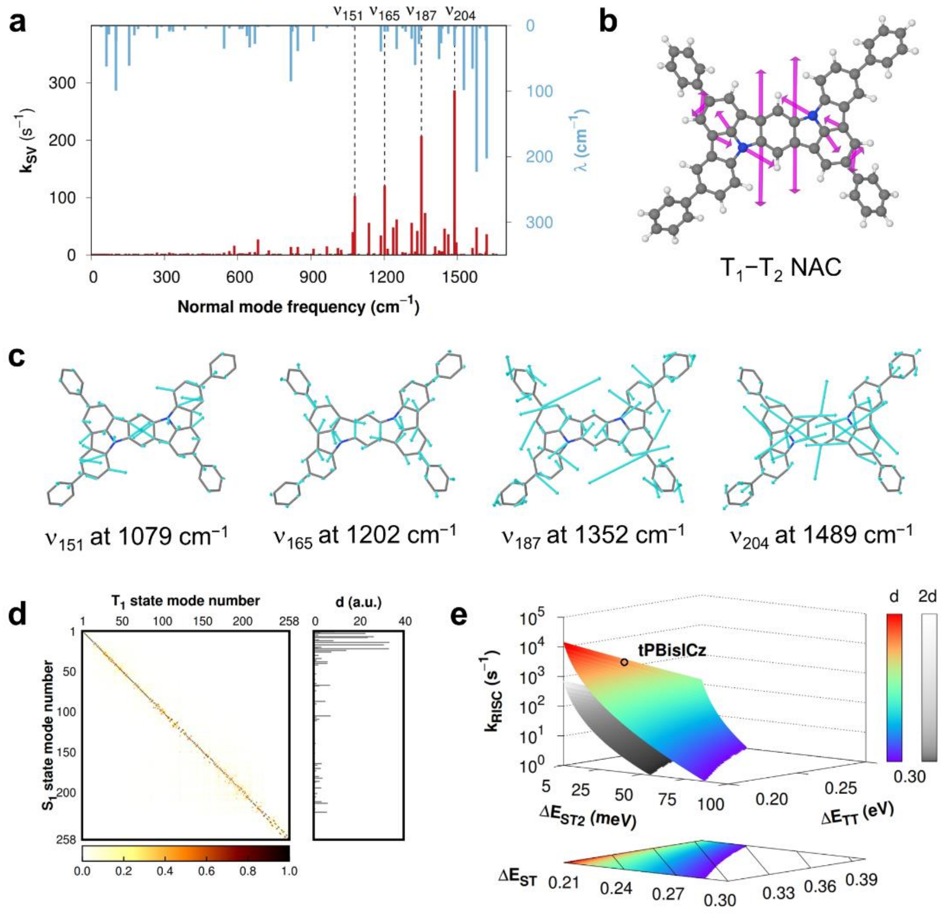

Supplementary Figure 9. Analysis of RISC dynamics. **a**,Deconvolution of SVC-RISC rate and reorganisation energyλ for each mode. Major contributing modes are marked as vertical lines.**b**,T_1–T_2 nonadiabatic coupling (NAC) vectors.**c,** Important normal vibrations of **tPBisICz** in the S_1 state. Hydrogen atoms are omitted forvisual clarity.**d**, Duschinsky (mode-mixing) matrix and displacement vectors related to the RISC transition. The elements are displayed with their absolute values. **e**, Surface plot (top) and contour plot (bottom) ofk_{RISC} simulated as a function of ΔE_{TT} and ΔE_{ST2} for **tPBisICz**. The contour plot is identical to Fig. 4b. The gray surface is drawn with twice the magnitude of the original displacement vectors.

1.4. Device fabrication and performances

The OLED devices were fabricated using a transparent glass substrate with 50 nm thick indium tin oxide (ITO). The ITO glass substrate was cleaned using acetone and deionised water by ultrasonic treatment. The optimised device structure is PEDOT:PSS (40 nm)/TAPC (5 nm)/TCTA (5 nm)/PCZAC (5 nm)/mCP (5 nm)/mCP:TSPO1:**BisICz/tBisICz/tPBisICz** (25 nm, 50%:1%)/TSPO1 (25 nm)/LiF (1.5 nm)/Al (200 nm).Here, PEDOT:PSS [poly(3,4-ethylenedioxythiophene)-poly(styrenesulfonate)] is a hole injection layer, TAPC [(1,1-bis(4-di-*p*-tolylaminophenyl)cyclohexane)], TCTA [tris(4-carbazoyl-9-ylphenyl)amine], and PCZAC [9,9-dimethyl-10-(9-phenyl-9*H*-carbazol-3-yl)-9,10-dihydroacridine] are hole transporting layers, and mCP [1,3-di(9*H*-carbazol-9-yl)benzene] is a hole-type exciton blocking layer. In the emitting layer, mCP was used as a hole-type host and TSPO1 [4-(triphenylsilyl)phenyldiphenyl phosphine oxide] was used as an electron-type host. TSPO1 was also used as an electron-type exciton blocking layer. All the layers were deposited by vacuum thermal evaporation under a pressure of 3.0×10^{-7}Torr. The deposition rates for the organic layers, LiF, and Al were controlled at 0.1, 0.01, and 0.5 nm/s, respectively. The encapsulation on glass was performed under an inert atmosphere (N_2 atmosphere) to protect the device from O_2. All the device measurements were performed at room temperature.

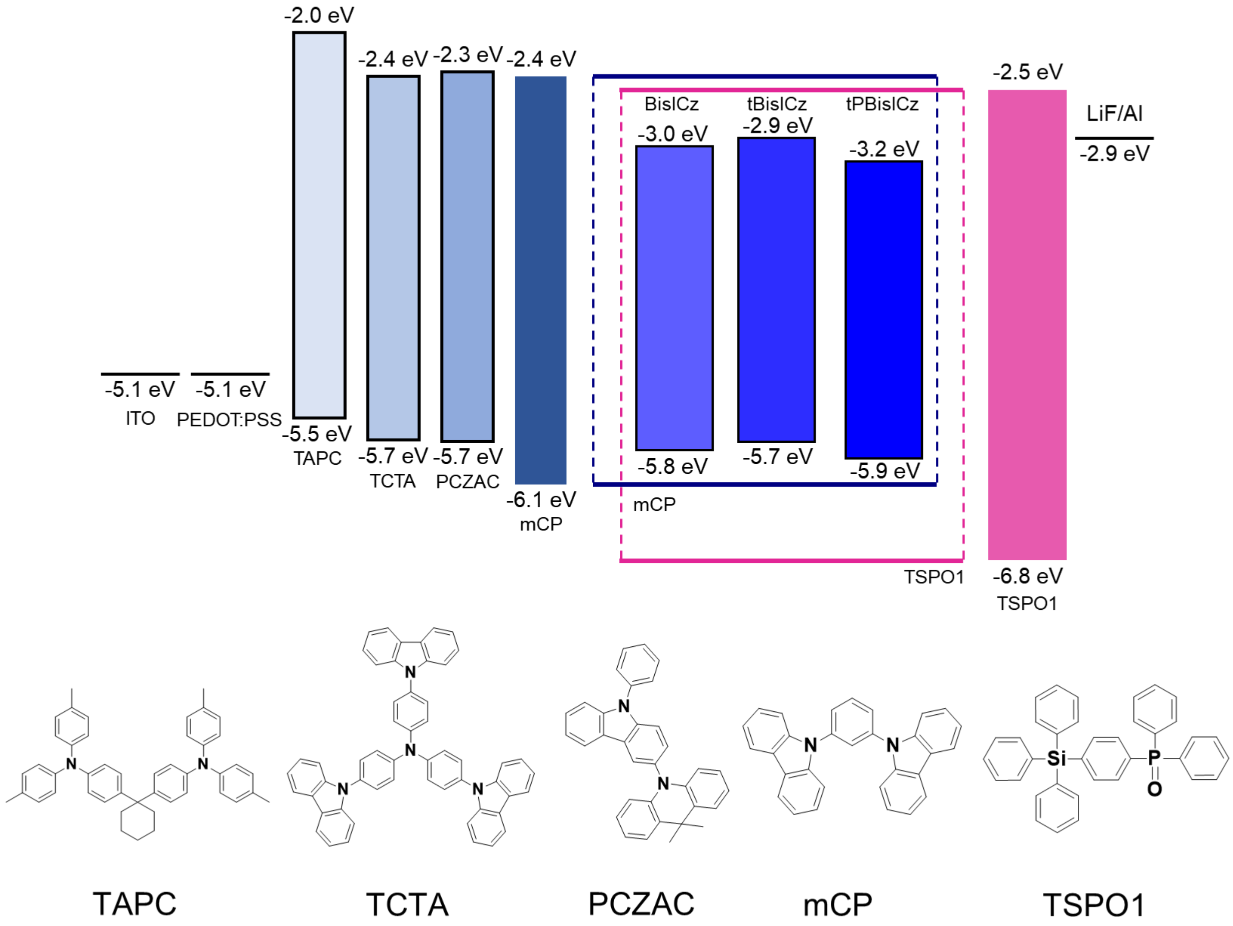

Supplementary Figure 10. Schematic device diagram of BisICz-, tBisICz-, and tPBisICz-doped devices and chemical structures.

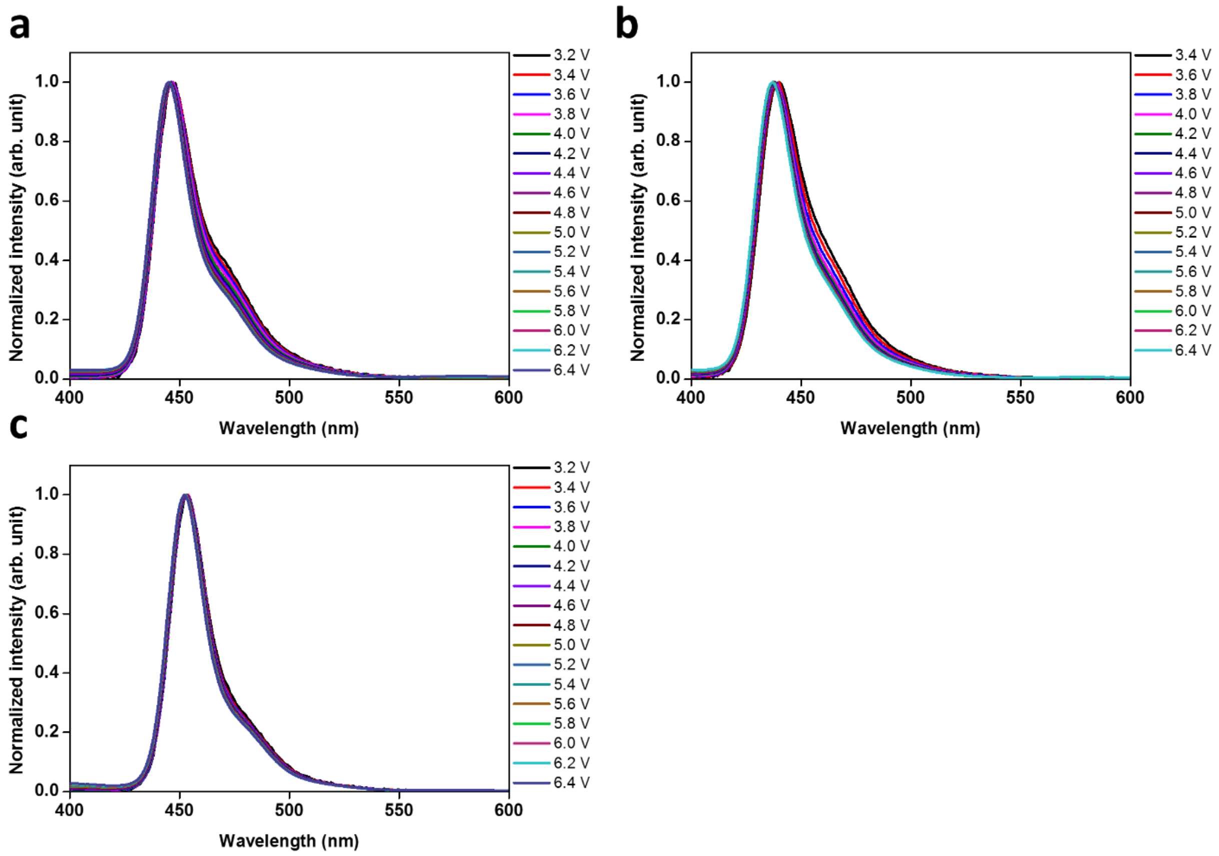

Supplementary Figure 11. Voltage-dependent electroluminescent spectra. **a**,**BisICz**, **b**,**tBisICz**, and **c**,**tPBisICz**.

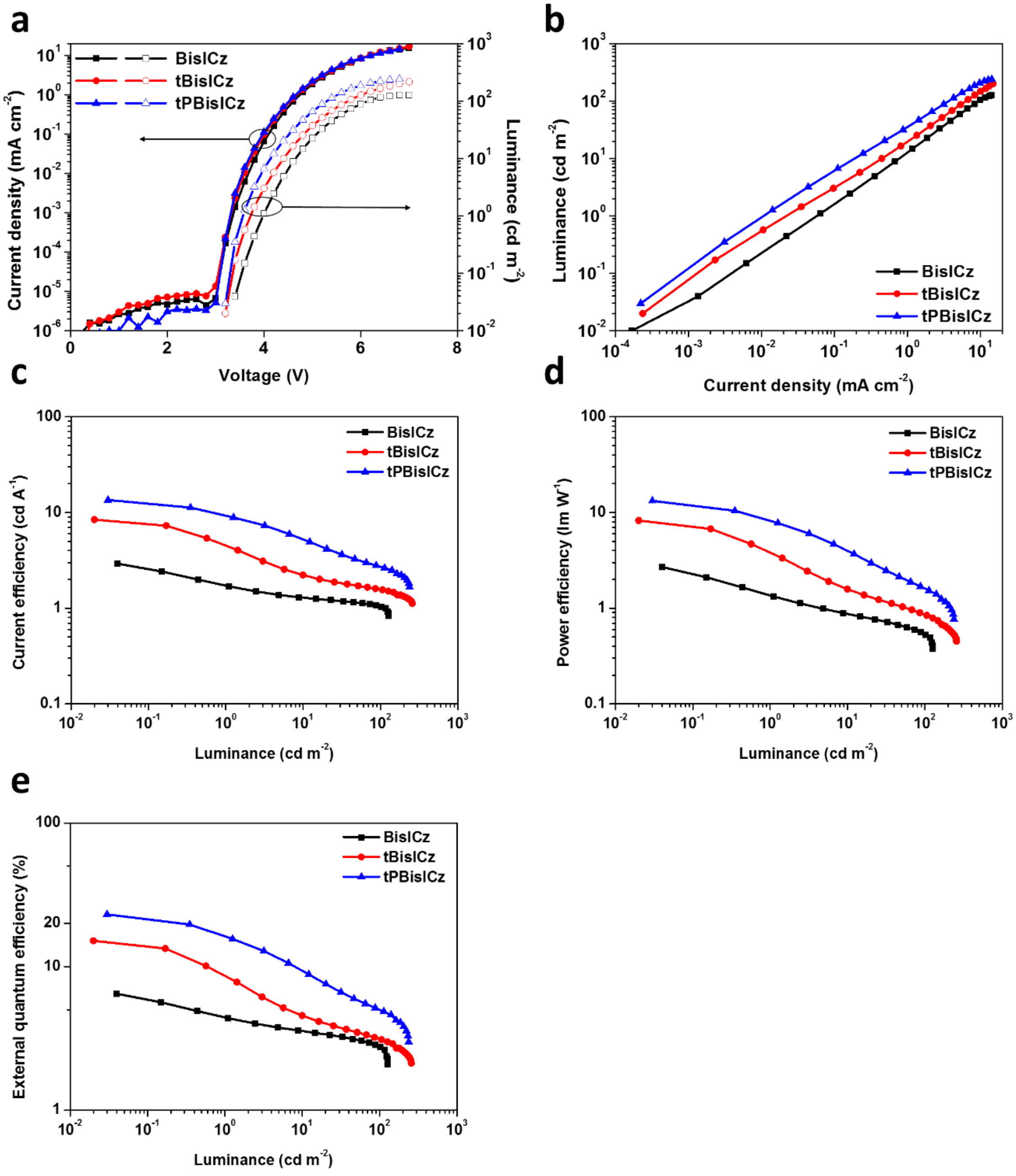

Supplementary Figure 12. Device performance. **a**,Current density-voltage-luminance, **b**,luminance-current density, **c**,currentefficiency-luminance, **d**, power efficiency-luminance, and **e**, external quantum efficiency-luminance curves of **BisICz**, **tBisICz**, and **tPBisICz**.

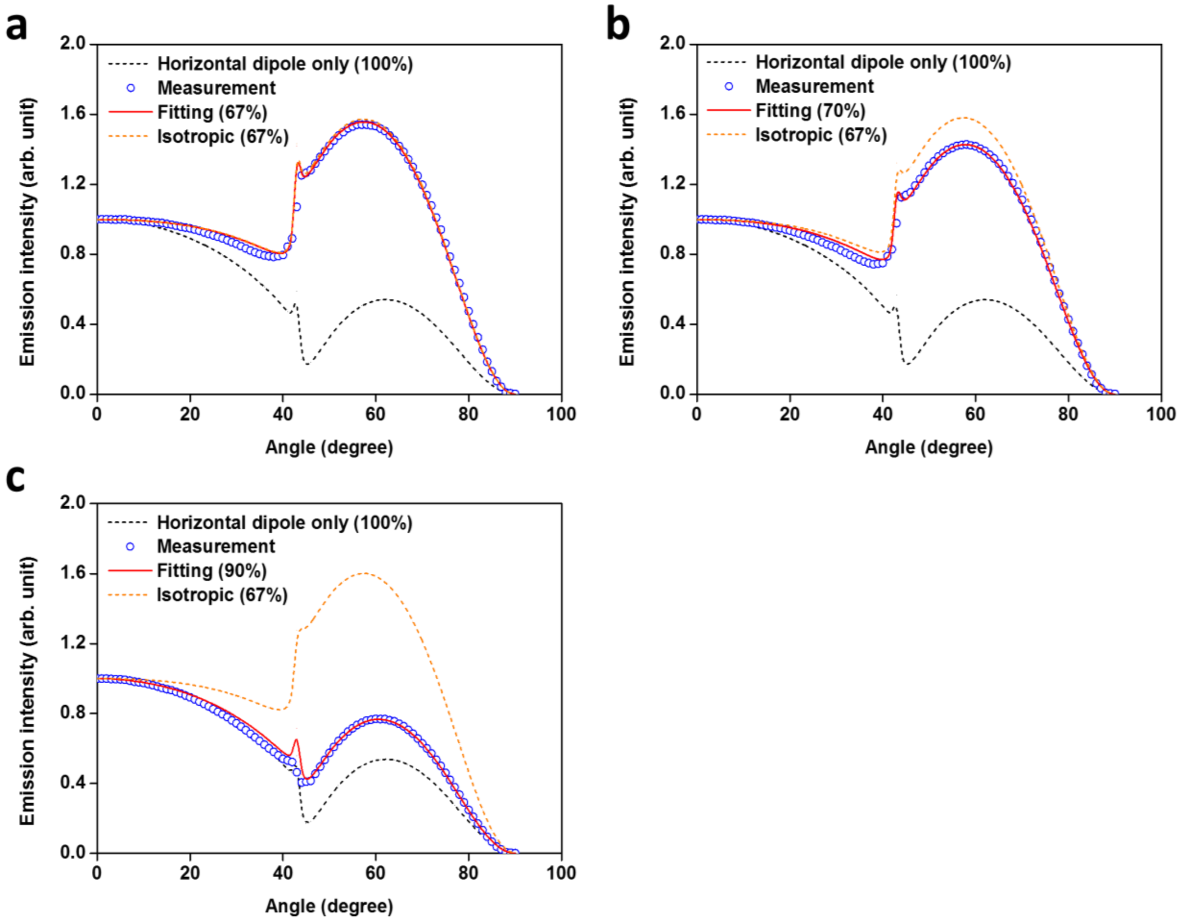

Supplementary Figure 13. Horizontal transition dipole moment ratios. **a**,**BisICz**, **b**,**tBisICz**, and **c**,**tPBisICz**.

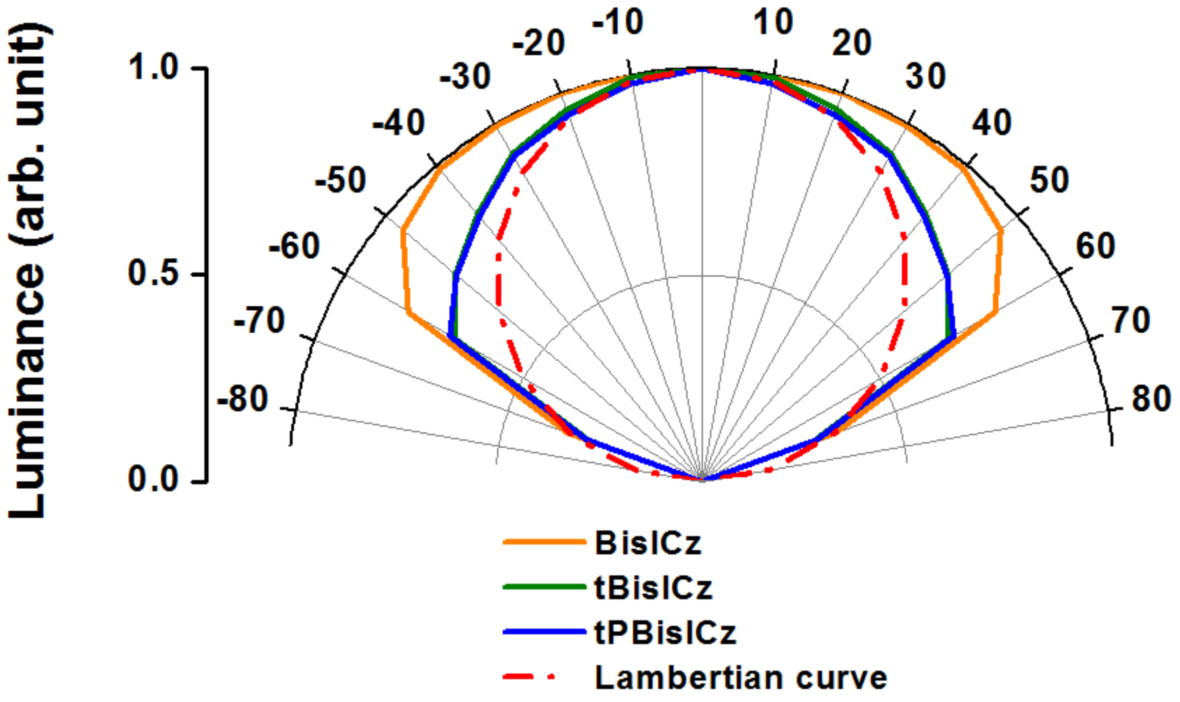

Supplementary Figure 14. The comparison EL data with Lambertian distribution.

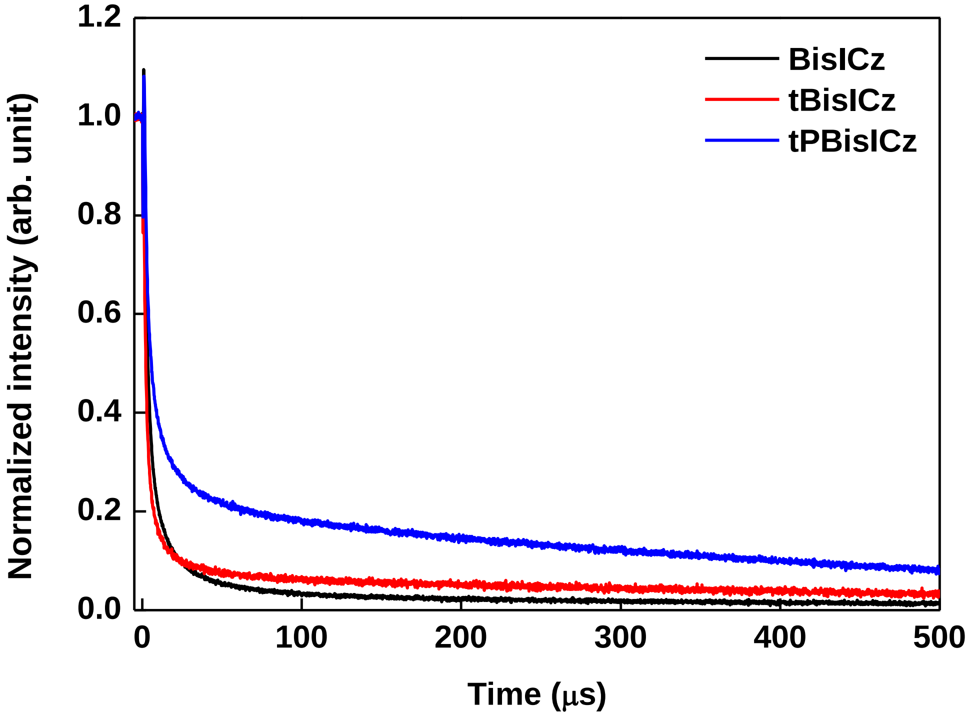

Supplementary Figure 15. The transient EL decay curves of fabricated devices.

SUPPLEMENTARY TABLES

Supplementary Table 1. Orbital energies, singlet and triplet energies and ΔE_{ST} of various molecules[a]

	**HOMO**[b]	**LUMO**[b]	E_S[c]	E_T[c]	ΔE_{ST}[c]
Triphenylamine derivatives					
**TPA**	−6.72	1.09	4.12	3.41	0.70
**PCz**	−7.21	0.76	3.99	3.40	0.59
**ICz**	−7.43	0.11	3.75	3.20	0.56
3,6-Substituted carbazoles					
**Cz**	−7.56	0.90	4.08	3.41	0.67
**Cz-*t*Bu**	−7.10	0.84	4.01	3.37	0.64
**Cz-Ph**	−7.02	0.54	3.90	3.39	0.51

[a]Energies are given in eV. [b]Calculated at the ω*B97M-V level using the S_1 geometry optimized at theSCS-ADC(2) level.[c]Adiabatic energies.

Supplementary Table 2. Calculation data for BisICz-based materials

**Compound**	**HOMO (eV)** [a]	**LUMO (eV)** [a]	ΔE_{ST} **(eV)** [b]	ΔE_{TT} **(eV)** [c]	**SOC (cm^{-1})** [d] S_1-T_1	**SOC (cm^{-1})** [d] S_1-T_2	f[e]	k_{RISC} **($10^3 s^{-1}$)**[f]
**BisICz**	−6.69	−0.54	0.33	0.218	0.224	0.675	0.102	0.008
**tBisICz**	−6.31	−0.54	0.261	0.287	0.144	0.578	0.122	0.27 (0.970)
**tPBisICz**[g]	−6.39	−0.79	0.249	0.243	0.096	0.554	0.170	1.47(0.999)

[a] Calculated at the ω*B97M-V level using the S_1 geometry optimized at the SCS-ADC(2) level. [b] Adiabatic singlet-triplet energy difference at the SCS-ADC(2) level. [c] Vertical triplet-triplet energy differences calculated at the ω*B97M-V level using the S_1 geometry. [d] Triplet substate-averaged spin–orbit coupling. [e] Oscillator strength. [f] Determined from the analytical solution of the Fermi's golden rule expression. Spin–vibronic contribution to total k_{RISC} is given in parentheses. [g] Phenyl groups were employed in place of di-3,5-*tert*-butylphenyl for computational ease.

Supplementary Table 3. Photophysical characteristics of ultrapure blue BisICz-based emitters

	**in 10^{-5} M DCM solution**					**in 1 wt%-doped mCP:TSPO1 film**						
**Compound**	λ_{PL} **(nm)**[a]	E_S/E_T **(eV)**[b]	ΔE_{ST} **(eV)**	**Stokes (nm)**[c]	**FWHM (nm)**	λ_{PL} **(nm)**[a]	E_S **(eV)**[b]	**Stokes (nm)**[d]	**FWHM (nm)**	**PLQY (%)**[e]	**PLQY (%)**[f]	k_{RISC} **($10^3 s^{-1}$)**
**BisICz**	429	2.89/ 2.58	0.31	15	23	436	2.84	22	27	76	81	-
**tBisICz**	436	2.84/2.55	0.29	12	22	442	2.80	18	21	77	95	0.15
**tPBisICz**	445	2.81/ 2.54	0.27	10	27	450	2.75	15	21	78	91	1.41

[a] PL maximum; excitation wavelength of 330 nm. [b] Estimated from maximum peak position of fluorescence (300 K) or phosphorescence (77 K). [c] Difference between lowest absorption peak in 10^{-5} M DCM solution and maximum emission peak from low-temperature fluorescence spectrum. [d] Difference between lowest absorption peak in 10^{-5} M DCM solution and maximum emission peak from film emission spectrum. [e] Absolute PL quantum yield; determined using integrating sphere under air atmosphere. [f]determined using integrating sphere under nitrogen atmosphere [g] Estimated according to method reported in [S16].

Supplementary Table 4. Transient PL quantum yields and rate constant calculations for BisICz, tBisICz, and tPBisICz.

	τ_{PF}[a] (ns)	k_{PF}[b] (10^7 s^{-1})	τ_{DF}[c] (ms)	k_{DF}[d] (10^2 s^{-1})	Φ_{PF}[e] (%)	Φ_{DF}[f] (%)	k_{ISC}[gh] (10^7 s^{-1})	k_{RISC}[gi] (10^3 s^{-1})	k^s_r[gj] (10^7 s^{-1})	k^s_{nr}[gk] (10^6 s^{-1})
**BisICz**	18.6	5.38	N.A.[l]	N.A.	81	N.A.	N.A.	N.A.	4.35	10.2
**tBisICz**	16.0	6.25	12.5	8.33	48	47	3.25	0.15	3.00	1.58
**tPBisICz**	10.9	9.17	1.74	5.75	35	56	5.96	1.41	3.21	3.18

[a]Prompt fluorescence lifetime. [b]Rate constant for prompt fluorescence lifetime. [c]Delayed fluorescence lifetime. [d]Rate constant for delayed fluorescence lifetime. [e]Prompt and [f] delayed PLQY measured in mCP:TSPO1: 1 wt%-doped thin film and calculated from total PLQY and ratio of integrated area of individual components in transient spectra to total integrated area. [g]Calculated using equations reported in[S17].[h]Rate constant for intersystem crossing (ISC). [I] Rate constant for reverse intersystem crossing (RISC). [j]Radiative decay rate constant for one singlet. [k]Non-radiative decay rate constant for one singlet. [l] N.A. denotes that data were not assigned.

Supplementary Table 5. Transient PL fitting data

		Excitation wavelength (nm)	Emission wavelength (nm)	CHI	<τ_{avg}>	τ_1	τ_2	A_1	A_2
**BisICz**	Prompt	280	435	1.05596	18.617 ns	11.215 ns	22.164 ns	4623.70	4685.42
	Delayed	280	N.A.[a]	N.A.	N.A.	N.A.	N.A.	N.A.	N.A.
**tBisICz**	Prompt	280	444	1.02086	15.984 ns	11.295 ns	18.815 ns	4793.87	4765.32
	Delayed	280	444	1.11648	12.493 ms	1.160ms	12.701 ms	1.74046	8.64021
**tPBisICz**	Prompt	280	450	1.10243	10.893 ns	9.7246 ns	16.634 ns	8849.96	1052.54
	Delayed	280	450	1.18202	1.742ms	1.7420 ms	N.A.	20.1412	N.A.

[a] N.A. denotes that data were not assigned.

Supplementary Table 6. Device performance of ultrapure blue OLEDs

		maximum				
**Compound**	**λ_{EL}(nm)[a]**	**EQE (%)**	**PE (lm W^{-1})**	**CE (cd A^{-1})**	**CIE (x, y)[a]**	**FWHM (nm)[a]**
**BisICz**	437	6.5[b]	2.7	2.9	(0.16, 0.04)	24
**tBisICz**	445	15.1[c]	8.3	8.4	(0.16, 0.05)	22
**tPBisICz**	452	23.1[d]	13.3	13.5	(0.15, 0.05)	21

[a]Values at luminance of 100 cd m^{-2}. [b]Values at luminance of 0.04 cd m^{-2}.[c]Values at luminance of 0.02 cd m^{-2}.[d]Values at luminance of 0.03 cd m^{-2}.

SupplementaryTable 7. Summary of reported deep-blue OLED device performance

Type	Emitter	Emissive layer	CIE	FWHM (nm)[a]	Max EQE	Ref.
Phosphorescence	$Ir(fdpt)_3$	DPEPO:$Ir(fdpt)_3$	(0.15, 0.11)[b]	N.A.[c]	22.5%	**S18**
	$(TF)_2Ir(fptz)$	mCPPO1:$(TF)_2Ir(fptz)$	(0.147, 0.116)[d]	≤50	8.4%	**S19**
	fac-$Ir(pmp)_3$	*fac*-$Ir(pmp)_3$/TSPO1:*fac*-$Ir(pmp)_3$	(0.16, 0.09)[e]	N.A.	10.1%	**S20**
	$Ir(fpmi)_2(tfpypz)$	UGH2:CzSi:$Ir(fpmi)_2(tfpypz)$	(0.14, 0.10)[f]	N.A.	7.6%	**S21**
	$Ir(fppz)_2(dfbdp)$	CzSi:Emitter/UGH2:Emitter	(0.152, 0.110)[g]	N.A.	11.9%	**S22**
	$Ir(fbppz)_2(dfbdp)$		(0.155, 0.106)[g]	N.A.	11.7%	
	Ir1	TSPO1:Emitter	(0.16, 0.12)[g]	N.A.	8.6%	**S23**
	Ir3		(0.16, 0.08)[g]	N.A.	7.1%	
	PtON1-tBu	26mCPy:Emitter	(0.151, 0.098)	24	5.3%	**S24**
	PtON6-tBu		(0.147, 0.093)	30	10.9%	
	PtON7-dtb	TAPC:PO15:PtON7-dtb	(0.148, 0.079)	29	24.8%	
	2	DPEPO:2	(0.154, 0.052)	63	13.4%	**S25**
	mer-Ir1	TSPO1:*mer*-Ir1	(0.149, 0.085)[e]	N.A.	24.8%	**S26**
DA TADF	4TCzPhBN	DPEPO:4TCzPhBN	(0.15, 0.10)	60	7.5%	**S27**
	PXB-mIC	PPBI:PXB-mIC	(0.15, 0.08)	N.A.	12.5%	**S28**
	CzAZB	mCP:CzAZB	(0.14, 0.10)	51	5.5%	**S29**
	TDBA-Ac	PPBI:TDBA-Ac	(0.15, 0.06)[e]	48	21.5%	**S30**
MR-TADF	DABNA-1	mCBP:DABNA-1	(0.13, 0.09)	28	13.5%	**S16**
	B2	mCBP:B2	(0.13, 0.11)	37	18.3%	**S31**
	DABNA-NP-TB	DOBNA-Tol:DABNA-NP-TB	(0.14, 0.11)	33	19.5%	**S32**
	v-DABNA	DOBNA-OAr:*v*-DABNA	(0.12, 0.11)	18	34.4%	**S33**
**SVC-TADF**	**BisICz**	mCP:TSPO1:Emitter	(0.16, 0.04)[g]	24	6.5%	**This Work**
	**tBisICz**		(0.16, 0.05)[g]	22	15.1%	
	**tPBisICz**		(0.15, 0.05)[g]	21	23.1%	

[a]Values from electroluminescence spectrum.[b]Values at current density of 9 mA cm^{-2}.[c]N.A. denotes that data were not assigned.[d]Values at current density of 5 mA cm^{-2}. [e]Values at current density of 10 mA cm^{-2}. [f]Values at voltage of 8 V.[g] Values at luminance of 100 cd m^{-2}.

SUPPLEMENTARY REFFERENCES

[S1] S. G. Balasubramani, G. P. Chen, S. Coriani, M. Diedenhofen, M. S. Frank, Y. J. Franzke, F. Furche, R. Grotjahn, M. E. Harding, C. Hättig, A. Hellweg, B. Helmich-Paris, C. Holzer, U. Huniar, M. Kaupp, A. M. Khah, S. K. Khani, T. Müller, F. Mack, B. D. Nguyen, S. M. Parker, E. Perlt, D. Rappoport, K. Reiter, S. Roy, M. Rückert, G. Schmitz, M. Sierka, E. Tapavicza, D. P. Tew, C. van Wüllen, V. K. Voora, F. Weigend, A. Wodyński, J. M. Yu, *J. Chem. Phys.***2020**, *152,* 184107.

[S2] Y. Shao, Z. Gan, E. Epifanovsky, A. T.B. Gilbert, M. Wormit, J. Kussmann, A. W. Lange, A. Behn, J. Deng, X. Feng, D. Ghosh, M. Goldey, P. R. Horn, L. D. Jacobson, I. Kaliman, R. Z. Khaliullin, T. Kuś, A. Landau, J. Liu, E. I. Proynov, Y. M. Rhee, R. M. Richard, M. A. Rohrdanz, R. P. Steele, E. J. Sundstrom, H. L. Woodcock III, P. M. Zimmerman, D. Zuev, B. Albrecht, E. Alguire, B. Austin, G. J. O. Beran, Y. A. Bernard, E. Berquist, K. Brandhorst, K. B. Bravaya, S. T. Brown, D. Casanova, C.-M. Chang, Y. Chen, S. H. Chien, K. D. Closser, D. L. Crittenden, M. Diedenhofen, R. A. DiStasio Jr., H. Do, A. D. Dutoi, R. G. Edgar, S. Fatehi, L. Fusti-Molnar, A. Ghysels, A. Golubeva-Zadorozhnaya, J. Gomes, M. W.D. Hanson-Heine, P. H.P. Harbach, A. W. Hauser, E. G. Hohenstein, Z. C. Holden, T.-C. Jagau, H. Ji, B. Kaduk, K. Khistyaev, J. Kim, J. Kim, R. A. King, P. Klunzinger, D. Kosenkov, T. Kowalczyk, C. M. Krauter, K. U. Lao, A. D. Laurent, K. V. Lawler, S. V. Levchenko, C. Y. Lin, F. Liu, E. Livshits, R. C. Lochan, A. Luenser, P. Manohar, S. F. Manzer, S.-P. Mao, N. Mardirossian, A. V. Marenich, S. A. Maurer, N. J. Mayhall, E. Neuscamman, C. M. Oana, R. Olivares-Amaya, D. P. O'Neill, J. A. Parkhill, T. M. Perrine, R. Peverati, A. Prociuk, D. R. Rehn, E. Rosta, N. J. Russ, S. M. Sharada, S. Sharma, D. W. Small, A. Sodt, T. Stein, D. Stück, Y.-C. Su, A. J.W. Thom, T. Tsuchimochi, V. Vanovschi, L. Vogt, O. Vydrov, T. Wang, M. A. Watson, J. Wenzel, A. White,

C. F. Williams, J. Yang, S. Yeganeh, S. R. Yost, Z.-Q. You, I. Y. Zhang, X. Zhang, Y. Zhao, B. R. Brooks, G. K.L. Chan, D. M. Chipman, C. J. Cramer, W. A. Goddard III, M. S. Gordon, W. J. Hehre, A. Klamt, H. F. Schaefer III, M. W. Schmidt, C. D. Sherrill, D. G. Truhlar, A. Warshel, X. Xu, A. Aspuru-Guzik, R. Baer, A. T. Bell, N. A. Besley, J.-D. Chai, A. Dreuw, B. D. Dunietz, T. R. Furlani, S. R. Gwaltney, C.-P. Hsu, Y. Jung, J. Kong, D. S. Lambrecht, W. Liang, C. Ochsenfeld, V. A. Rassolov, L. V. Slipchenko, J. E. Subotnik, T. Van Voorhis, J. M. Herbert, A. I. Krylov, P. M. W. Gill, M. Head-Gordon, *Mol. Phys.***2015**, *113*, 184.

[S3] M. Gerenkamp, S. Grimme, *Chem. Phys. Lett.***2004**, *392*, 229.

[S4] J. Schirmer, *Phys. Rev. A***1982**, *26*, 2395.

[S5] D. H. Friese, L. Törk, C. Hättig, *J. Chem. Phys.***2014**, *141*, 194106.

[S6] N. Mardirossian, M. Head-Gordon, *J. Chem. Phys.***2016**, *144*, 214110.

[S7] H. Sun, C. Zhong, J.-L. Brédas, *J. Chem. Theory Comput.***2015**, *11*, 3851.

[S8] J. Tomasi, B. Mennucci, R. Cammi, *Chem. Rev.***2005**, *105*, 2999.

[S9] F. Weigend, R. Ahlrichs, *Phys. Chem. Chem. Phys.***2005**, *7*, 3297.

[S10] Q. Peng, Y. Niu, Q. Shi, X. Gao, Z. Shuai, *J. Chem. Theory Comput.***2013**, *9*, 1132.

[S11] A. Baiardi, J. Bloino, V. Barone, *J. Chem. Theory Comput.***2013**, *9*, 4097.

[S12] B. de Souza, G. Farias, F. Neese, R. Izsák, *J. Chem. Theory Comput.***2019**, *15*, 1896.

[S13] I. Kim, S. O. Jeon, D. Jeong, H. Choi, W.-J. Son, D. Kim, Y. M. Rhee, H. S. Lee, *J. Chem. Theory Comput.***2020**, *16*, 621.

[S14] J. R. Reimers, *J. Chem. Phys.***2001**, *115*, 9103.

[S15] F. Plasser, M. Wormit, A. Dreuw, *J. Chem. Phys.***2014**, *141*, 024106.

[S16] T. Hatakeyama, K. Shiren, K. Nakajima, S. Nomura, S. Nakatsuka, K. Kinoshita, J. Ni, Y. Ono, T. Ikuta, *Adv. Mater.***2016**, *28*, 2777–2781.

[S17] G. H. Kim, R. Lampande, J. B. Im, J. Y. Lee, J. H. Kwon, *Mater. Horiz.* **2017**, *4*, 619–624.

[S18] X. Li, J. Zhang, Z. Zhao, L. Wang, H. Yang, Q. Chang, N. Jiang, Z. Liu, Z. Bian, W. Liu, Z. Lu, C. Huang, *Adv. Mater.***2018**, *30*, 1705005.

[S19] S. Lee, S.-O. Kim, H. Shin, H.-J. Yun, K. Yang, S.-K. Kwon, J.-J. Kim, Y.-H. Kim, *J. Am. Chem. Soc.***2013**, *135*, 14321.

[S20] J. Lee, H.-F. Chen, T. Batagoda, C. Coburn, P. I. Djurovich, M. E. Thompson, S. R. Forrest, *Nat. Mater.***2016**, *15*, 928.

[S21] C. H. Hsieh, F.-I. Wu, C.-H. Fan, M.-J. Huang, K.-Y. Lu, P.-Y. Chou, Y.-H. O. Yang, S.-H. Wu, I.-C. Chen, S.-H. Chou, K.-T. Wong, C.-H. Cheng, *Chem. Eur. J.***2011**, *17*, 9180.

[S22] Y. C. Chiu, J.-Y. Hung, Y. Chi, C.-C. Chen, C.-H. Chang, C.-C. Wu, Y.-M. Cheng, Y.-C. Yu, G.-H. Lee, P.-T. Chou, *Adv. Mater.***2009**, *21*, 2221.

[S23] G. Sarada, A. Maheshwaran, W. Cho, T. Lee, S. H. Han, J. Y. Lee, S.-H. Jin, *Dyes Pigm.***2018**, *150*, 1.

[S24] T. Fleetham, G. Li, L. Wen, J. Li, *Adv. Mater.***2014**, *26*, 7116.

[S25] A. K. Pal, S. Krotkus, M. Fontani, C. F. R. Mackenzie, D. B. Cordes, A. M. Z. Slawin, I. D. W. Samuel, E. Zysman-Colman, *Adv. Mater.***2018**, *30*, 1804231.

[S26] H.-Y. Park, A. Maheshwaran, C.-K. Moon, H. Lee, S. S. Reddy, V. G. Sree, J. Yoon, J. W. Kim, J. H. Kwon, J.-J. Kim, S.-H. Jin, *Adv. Mater.***2020**, *32*, 2002120.

[S27] D. Zhang, X. Song, A. J. Gillett, B. H. Drummond, S. T. E. Jones, G. Li, H. He, M. Cai, D. Credgington, L. Duan, *Adv. Mater.***2020**, *32*, 1908355.

[S28] D. H. Ahn, H. Lee, S. W. Kim, D. Karthik, J. Lee, H. Jeong, J. Y. Lee, J. H. Kwon, *ACS Appl. Mater. Interfaces***2019**, *11*, 14909–14916.

[S29] T.-L. Wu, S.-H. Lo, Y.-C. Chang, M.-J. Huang, C.-H. Cheng, *ACS Appl. Mater. Interfaces*, **2019**, *11*, 10768–10776.

[S30] D. H. Ahn, S. W. Kim, H. Lee, I. J. Ko, D. Karthik, J. Y. Lee, J. H. Kwon, *Nat. Photonics* **2019**, *13*, 540–546.

[S31] K. Matsui, S. Oda, K. Yoshiura, K. Nakajima, N. Yasuda, T. Hatakeyama, *J. Am. Chem. Soc.* **2018**, *140*, 1195–1198.

[S32] S. Oda, W. Kumano, T. Hama, R. Kawasumi, K. Yoshiura, T. Hatakeyama, *Angew. Chem. Int. Ed.* **2020**, *133*, 2918–2922.

[S33] Y. Kondo, K. Yoshiura, S. Kitera, H. Nishi, S. Oda, H. Gotoh, Y. Sasada, M. Yanai, T. Hatakeyama, *Nat. Photonics* **2019**, *13*, 678–682.
